# Towards a scientific community consensus on designating Vulnerable Marine Ecosystems from imagery

**DOI:** 10.7717/peerj.16024

**Published:** 2023-10-12

**Authors:** Amy R. Baco, Rebecca Ross, Franziska Althaus, Diva Amon, Amelia E. H. Bridges, Saskia Brix, Pål Buhl-Mortensen, Ana Colaco, Marina Carreiro-Silva, Malcolm R. Clark, Cherisse Du Preez, Mari-Lise Franken, Matthew Gianni, Genoveva Gonzalez-Mirelis, Thomas Hourigan, Kerry Howell, Lisa A. Levin, Dhugal J. Lindsay, Tina N. Molodtsova, Nicole Morgan, Telmo Morato, Beatriz E. Mejia-Mercado, David O’Sullivan, Tabitha Pearman, David Price, Katleen Robert, Laura Robson, Ashley A. Rowden, James Taylor, Michelle Taylor, Lissette Victorero, Les Watling, Alan Williams, Joana R. Xavier, Chris Yesson

**Affiliations:** 1 Earth, Ocean, and Atmospheric Sciences, Florida State University, Tallahassee, FL, United States; 2 Institute of Marine Research, Bergen, Norway; 3 CSIRO Environment, Hobart, Australia; 4 SpeSeas, D’Abadie, Trinidad and Tobago; 5 Marine Science Institute, University of California, Santa Barbara, Santa Barbara, California, United States; 6 School of Biological and Marine Science, University of Plymouth, Plymouth, United Kingdom; 7 Senckenberg am Meer, German Center for Marine Biodiversity Research (DZMB), Senckenberg Nature Research Society, Hamburg, Germany; 8 Okeanos-University of the Azores, Horta, Portugal; 9 National Institute of Water & Atmospheric Research, Wellington, New Zealand; 10 Fisheries and Oceans Canada, Sidney, Canada; 11 University of Victoria, Victoria, British Columbia, Canada; 12 University of Cape Town, Cape Town, South Africa; 13 Deep-Sea Conservation Coalition, Amsterdam, Netherlands; 14 National Oceanic & Atmospheric Administration, Washington, D.C., United States; 15 Scripps Institution of Oceanography, University of California, San Diego, California, United States; 16 Japan Agency for Marine-Earth Science and Technology, Yokosuka, Japan; 17 Shirshov Institute of Oceanology RAS, Moscow, Russia; 18 INFOMAR & Marine Institute, Galway, Ireland; 19 South Atlantic Environmental Research Institute, Stanley, Falkland Islands; 20 The National Oceanography Centre, Southampton, United Kingdom; 21 University of Southampton, Southampton, United Kingdom; 22 Fisheries and Marine Institute of Memorial University, St. John’s, Canada; 23 Joint Nature Conservation Committee, Peterborough, United Kingdom; 24 Victoria University of Wellington, Wellington, New Zealand; 25 School of Life Sciences, University of Essex, Essex, United Kingdom; 26 Norwegian Institute for Water Research, Bergen, Norway; 27 University of Aveiro, CESAM, Aveiro, Portugal; 28 University of Hawaii at Manoa, Honolulu, United States; 29 Department of Biological Sciences, University of Bergen, Bergen, Norway; 30 CIIMAR, Interdisciplinary Centre of Marine and Environmental Research, CIIMAR, University of Porto, Matsosinhos, Portugal; 31 Zoological Society of London, London, United Kingdom

**Keywords:** Vulnerable marine ecosystems, Significant adverse impacts, Areas beyond national jurisdiction, Deep-Sea imagery, VME indicator taxa

## Abstract

Management of deep-sea fisheries in areas beyond national jurisdiction by Regional Fisheries Management Organizations/Arrangements (RFMO/As) requires identification of areas with Vulnerable Marine Ecosystems (VMEs). Currently, fisheries data, including trawl and longline bycatch data, are used by many RFMO/As to inform the identification of VMEs. However, the collection of such data creates impacts and there is a need to collect non-invasive data for VME identification and monitoring purposes. Imagery data from scientific surveys satisfies this requirement, but there currently is no established framework for identifying VMEs from images. Thus, the goal of this study was to bring together a large international team to determine current VME assessment protocols and establish preliminary global consensus guidelines for identifying VMEs from images. An initial assessment showed a lack of consistency among RFMO/A regions regarding what is considered a VME indicator taxon, and hence variability in how VMEs might be defined. In certain cases, experts agreed that a VME could be identified from a single image, most often in areas of scleractinian reefs, dense octocoral gardens, multiple VME species’ co-occurrence, and chemosynthetic ecosystems. A decision flow chart is presented that gives practical interpretation of the FAO criteria for single images. To further evaluate steps of the flow chart related to density, data were compiled to assess whether scientists perceived similar density thresholds across regions. The range of observed densities and the density values considered to be VMEs varied considerably by taxon, but in many cases, there was a statistical difference in what experts considered to be a VME compared to images not considered a VME. Further work is required to develop an areal extent index, to include a measure of confidence, and to increase our understanding of what levels of density and diversity correspond to key ecosystem functions for VME indicator taxa. Based on our results, the following recommendations are made: 1. There is a need to establish a global consensus on which taxa are VME indicators. 2. RFMO/As should consider adopting guidelines that use imagery surveys as an alternative (or complement) to using bycatch and trawl surveys for designating VMEs. 3. Imagery surveys should also be included in Impact Assessments. And 4. All industries that impact the seafloor, not just fisheries, should use imagery surveys to detect and identify VMEs.

## Introduction

Management of fisheries in Areas Beyond National Jurisdiction (ABNJ, also referred to as the ‘high seas’) requires consideration of the potential or actual impact on vulnerable marine ecosystems (VMEs) under United Nations General Assembly (UNGA) resolutions 59/25 (UNGA Resolution 60/31, 2005), 61/105 (UNGA Resolution 61/105, 2007) and subsequent resolutions. VME is a term adopted by the UNGA to refer to areas where benthic ecosystems vulnerable to damage from bottom fishing exist or are likely to occur. The multilaterally agreed UN FAO International Guidelines for the Management of Deep-Sea Fisheries in the High Seas (FAO, 2009), hereinafter referred to as the FAO Guidelines, were adopted in 2008 and subsequently endorsed by UNGA resolution 64/72 (UNGA Resolution 65/38, 2010). These FAO Guidelines establish internationally agreed criteria for the identification of deep-sea areas, species and communities on the high seas that are particularly vulnerable to human impacts, and slow to recover from such impacts, specifically in reference to potential impacts from bottom contact fisheries. The general approach to VME designation in the FAO Guidelines is defined as:

“14. Vulnerability is related to the likelihood that a population, community, or habitat will experience substantial alteration from short-term or chronic disturbance, and the likelihood that it would recover and in what time frame. These are, in turn, related to the characteristics of the ecosystems themselves, especially biological and structural aspects. VME features may be physically or functionally fragile. The most vulnerable ecosystems are those that are both easily disturbed and very slow to recover, or may never recover.

15. The vulnerability of populations, communities and habitats must be assessed relative to specific threats. Some features, particularly those that are physically fragile or inherently rare, may be vulnerable to most forms of disturbance, but the vulnerability of some populations, communities and habitats may vary greatly depending on the type of fishing gear used or the kind of disturbance experienced.

16. The risks to a marine ecosystem are determined by its vulnerability, the probability of a threat occurring and the mitigation means applied to the threat,” (FAO, 2009).

Specific criteria for the identification of VMEs are presented in paragraph 42 of the FAO Guidelines, including: uniqueness or rarity, functional significance, fragility, life history traits that contribute to slow recovery, and areas of structural complexity (Table 1) (FAO, 2009). Only one of these criteria needs to be met for a site to be designated as a VME (FAO, 2009). Annex 1 of the guidelines contains a non-exhaustive list of the types of species, species groups, communities, and habitats that may contribute to forming VMEs, and also provides examples of topographical, hydrophysical or geological features that potentially support VMEs (*e.g*., summits and flanks of seamounts, canyons). Similar designations to VMEs, such as the Convention on Biological Diversity’s Ecologically and Biologically Significant Areas (EBSAs), OSPAR Threatened and/or Declining Species and Habitats, *etc*., have been established for ABNJ and by many States for their national waters (see Box 1). The broad range of independent international efforts to classify seafloor ecosystems and to recognize the threats to particularly vulnerable seafloor ecosystems emphasizes the importance being placed on their protection.

**Table 1:** Criteria for designating a VME from UN Food and Agriculture Organization (FAO).

FAO criteria	Definition	Examples
Uniqueness or rarity	*“an area or ecosystem that is unique or that contains rare species whose loss could not be compensated for by similar areas or ecosystems”*	Hydrothermal vents are home to hundreds of endemic molluscs, the majority of which are Critically Endangered, Endangered, or Vulnerable for extinction risk on the IUCN Red List (Thomas et al., 2021).
Functional significance of the habitat	*“discrete areas or habitats* *that are necessary for the survival, function, spawning/reproduction or recovery of fish stocks, particular life- history stages (*e.g*. nursery grounds or rearing areas), or of rare, threatened or endangered marine species”*	Sponge grounds of the Schulz bank as nursery for the Arctic skate *Amblyraja hyperborea* (Meyer et al., 2019) Cold-water coral reefs in the NE Atlantic are used as spawning grounds for the blackmouth catshark *Galeus melastomus* (Henry et al., 2013)
Fragility	*“an ecosystem that is highly susceptible to degradation by anthropogenic activities”*	The brittle skeletons of glass sponges cannot withstand mechanical stress and break and crumble (Krautter et al., 2001); The 3-dimensional structure of the matrix (reef) forming scleractinian *Solenosmilia variabilis* is susceptible to breaking into clumps and rubble by mechanical stress (Williams et al., 2020b)
Life-history traits of component species that make recovery difficult	*“ecosystems that are characterized by populations or assemblages of species with one or more of the following characteristics:* – *slow growth rates;* – *late age of maturity;* – *low or unpredictable recruitment; or* – *long-lived.”*	Jochum et al. (2012) estimated that a glass sponge spicule of *Monorhaphis chuni* had been growing for ~11,000 years; Deep-sea corals may live for thousands of years, with the oldest known specimen of a zoanthid aged to 4,265 years (Roark et al., 2009).
Structural complexity	*“an ecosystem that is characterized by complex physical structures created by significant concentrations of biotic and abiotic features… such ecosystems often have high diversity, which is dependent on the structuring organisms”*	Enhanced local epibenthic diversity in the presence of the structure-forming sponge *Vazella pourtalesii* (Hawkes et al., 2019); Diverse seamount communities in Australia and New Zealand are associated with matrix formed over geological times by the scleractinian *Solenosmilia variabilis* (Williams et al., 2010); Structural complexity within cold-water coral reefs found in the NE Atlantic influences biodiversity, species abundance, and fine-scale distribution of associated taxa (Price et al., 2019; Price et al., 2021)

**Note:**

Based on the FAO definition of VME: “A marine ecosystem should be classified as vulnerable based on the characteristics that it possesses. The following list of characteristics should be used as criteria in the identification of VMEs.” “(Paragraph 42, FAO DSF Guidelines).” http://www.fao.org/in-action/vulnerable-marine-ecosystems/criteria/fr/.

Key provisions of the UNGA resolutions and FAO Guidelines have largely been incorporated into management measures for bottom fisheries adopted by most Regional Fisheries Management Organizations or Arrangements (RFMO/As), that have the competence to manage bottom fisheries in ABNJ areas (*e.g*., Terje Løbach et al., 2020). In some cases, comparable provisions have also been incorporated in legislation for managing deep-sea bottom fisheries in areas within national waters (*e.g*., EU Regulation 2016/2336, Article 9, Box 1). These provisions establish three requirements for RFMO/As; the first is to identify areas where VMEs are “known or likely to occur”, the second is to determine whether one or more types of bottom fishing is causing or is likely to cause Significant Adverse Impacts (SAIs) on the VMEs, and the third is to manage bottom fisheries to prevent SAIs from occurring (*e.g*., Thompson et al., 2016).

To meet these three requirements, these provisions commit States and RFMO/As to conduct Impact Assessments (IAs) that include, *inter alia*, addressing “baseline information on the ecosystems, habitats and communities in the fishing area, against which future changes are to be compared” and the “identification, description and mapping of VMEs known or likely to occur in the fishing area” prior to authorizing, or continuing to authorize, bottom fishing in an area (UNGA Resolution 64/72, 2009, paragraph 119(a); FAO Guidelines, paragraph 47). Mapping should include compiling information on the distributions of likely VME indicators and habitats, creation of benthic feature or habitat maps, and predictive distribution models for VME indicators and habitats (Ardron et al., 2014).

**Figure 1:**
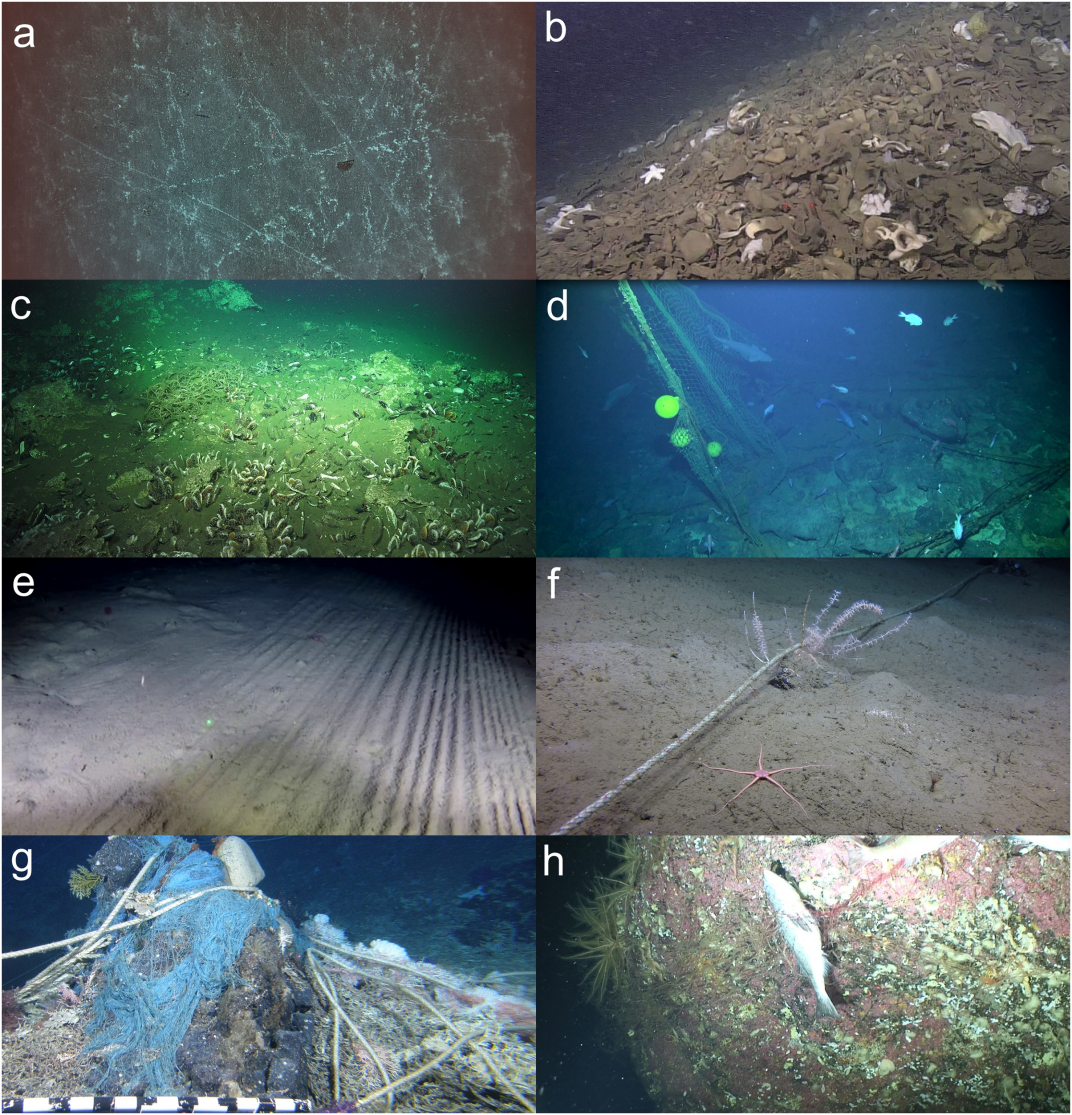
Examples of images that could be illustrative of significant adverse impacts (SAIs). (A) Barren seafloor on Yuryaku Seamount in the Emperor Seamount Chain showing multiple scars from bottom contact gear (Baco, Morgan & Roark, 2020, CC BY-NC-ND 4.0). (B) Sponge rubble from Learmonth bank, a granite knoll lying in EEZ waters of the border between Canada and Alaska (north of Haida Gwaii). The pile of dead glass sponges (family: Rossellidae) were (likely) detached/crushed from fishing gear, rolled around on the seafloor (which creates that distinct potato shape), and then accumulated against the base of Learmonth Bank because of the circulation patterns in Dixon Entrance. Image: Chu (2010) (C) lost fishing line at a Costa Rica methane seep (~1,000 m). Image: Schmidt Ocean Institute. FK190106, E. Cordes Chief Scientist. (D) A discarded trawl net and floats hooked on the seabed of a seamount off New Zealand at 900 m depth Image: NIWA. (E) Marks from demersal trawling over soft sediment habitats (~1,000 m depth) off Greenland (Long et al., 2021). (F) With an average set length of 3 km, derelict bottom longlines on Northeast Pacific Seamounts are extensive and fairly mobile, entangling and destroying biological structures while scouring the seafloor (Dellwood South Seamount). Image: Ocean Exploration Trust/Northeast Pacific Seamount Expedition Partners, J. Pegg (Fisheries and Oceans Canada). © His Majesty the King in Right of Canada, 2023 (G) image of lost fishing gear entangled in deep-sea corals on Southeast Hancock Seamount in the Northwestern Hawaiian Islands. Image: A. Baco FSU, and E.B. Roark TAMU, NSF, with HURL Pilots T. Kerby and M. Cremer. (H) Dead Widow Rockfish in a lost gill net on the summit of Cobb Seamount. Image: Curtis et al. (2015) /Fisheries and Oceans Canada. © His Majesty the King in Right of Canada, 2023.

In the FAO Guidelines, the criteria for determining whether impacts constitute SAIs are: “those that compromise ecosystem integrity (*i.e*., ecosystem structure or function) in a manner that: (i) impairs the ability of affected populations to replace themselves; (ii) degrades the long—term natural productivity of habitats; or (iii) causes, on more than a temporary basis, significant loss of species richness, habitat or community types”, with “temporary” to be determined on a case-by-case basis but which “should be in the order of 5–20 years” (FAO, 2009). Figure 1 includes examples of images of areas of seafloor that have experienced heavy impacts from bottom contact gear that could be illustrative of SAIs. Measures in place to protect VMEs from SAIs include the implementation of bottom-fishing closure areas, only permitting bottom fishing in areas where bottom fishing has previously occurred (the bottom fisheries’ ‘footprint’), or where an IA has demonstrated that fishing in the area would not cause SAIs to VMEs, and “move-on” rules for fishing vessels, related to encounters of specific quantities of VME indicator species or taxa (hereafter “VME indicators”) as bycatch from bottom-contacting gears.

Methods for determining the locations of VMEs, independent of fisheries collected data (*i.e*., bycatch), are being used by some RFMOs. For example, the North Atlantic Fisheries Organisation (NAFO) uses an approach of defining VMEs based on kernel density estimates of VME indicators derived from research vessel bottom trawl surveys (Kenchington et al., 2014); with species distribution models built from records of occurrences of VME indicators and imagery data is used to build on this approach (*e.g*., NAFO, 2014, 2019). In addition, the North-East Atlantic Fisheries Commission (NEAFC) uses a multi-criteria assessment approach based on a combination of imagery data, VME indicator database records, and a confidence index of the quality of observations (Morato et al., 2018).

However not all RFMOs employ such approaches, and even those that do continue to use ‘move-on rules’ for bottom contact fisheries as one of the main tools for defining VME areas (Table 2, reviewed in Walmsley et al., 2021). These rules require a fishing vessel to move a minimum distance away from the fishing location if a threshold level of bycatch of a VME indicator is encountered during the fishing activity (Auster et al., 2011). Encounter thresholds are typically based on bycatch data from trawls or longlines and are not designed for mapping areas of VMEs, but encounters at these threshold levels are instead used to indicate evidence of a VME (Auster et al., 2011). However, besides potentially destroying the VME in gathering these data, there are caveats to using bycatch to identify areas of VMEs. One of the biggest issues is that not all the impacted individuals are captured in fisheries gear, resulting in bycatch data that is not representative of what is on the seafloor (Wassenberg, Dews & Cook, 2002; Heifetz, Stone & Shotwell, 2009; Jones & Lockhart, 2011; Pitcher, Williams & Georgeson, 2019). For example, based on the approach of Freese et al. (1999) for calculating the area swept by a net, Auster et al. (2011) estimated the catch efficiency level of benthic gear for corals and sponges to be 1%. This means a 100-fold density over the weight threshold of VME indicators, such as corals and sponges, on the seafloor would be needed to trigger the move-on rule. Compositional biases are also introduced as the most fragile taxa, like xenophyophores or deep-sea corals, could be destroyed but not recovered in the gear, or may not be recovered in identifiable condition in bottom trawl bycatch (Pitcher, Williams & Georgeson, 2019). Indeed, although Jac et al. (2021) concluded that in certain environments there can be good parity between video and trawl sampling techniques, they found that in some environments the assumed dominant species may be different when sampled with imagery as compared to trawl surveys, with imagery capturing more erect, fragile species and trawls including more infauna.

**Table 2:** List of VME indicators by RFMO/A at time of manuscript submission. Taxonomy based on the World Register of Marine Species (https://www.marinespecies.org/).

	Qualifying taxon/Feature	Taxonomic rank	RFMO/A								
			NPFC	SPRFMO	NEAFC	NAFO ABNJ	CCAMLR	SEAFO	GFCM		SIOFA
Cnidaria	Actiniaria	Order	No	Yes	No	No	Yes	No	No		Yes
	Ceriantharia	Subclass	No	No	Yes (but only Cerianthidae)	Yes (but only Cerianthidae)	No	Yes (but only Cerianthidae)	Yes		No
	Alcyonacea*	Order	Yes (as Alcyonacea & Gorgonacea)	Yes (as “Gorgonian Alcyonacea (Holaxonia, Calaxonia, Scleraxonia”) & “Alcyonacea” (excluding gorgonians)”)	Yes	Yes (as Gorgonian Alcyonacea-suborders Holaxonia, Calaxonia, Scleraxonia)	Yes (as Alcyonacea & Gorgonacea)	Yes (as Alcyonacea & Gorgonacea)	Yes		Yes (as Alcyonacea & Gorgonacea)
	Pennatulacea	Order	No	Yes	Yes	Yes	Yes	Yes	Yes		Yes
	Antipatharia	Order	Yes	Yes	Yes (Schizopathidae, Leiopathidae, Antipathidae)	Yes	Yes	Yes	Yes		Yes
	Scleractinia	Order	Yes	Yes (as five genera genera: *Solenosmilia; Goniocorella; Oculina;* *Enallopsammia; Madrepora; Lophelia*)	Yes	Yes (Four branching spp. *E. rostrata, L. pertusa, M. oculata, S. variabilis*)	Yes	Yes	Yes		Yes
	Hydroidolina	Subclass	No	Yes (as the orders Anthoathecata & Leptothecata)	No	No	Yes	Yes (but only Antoathecatae)	Yes		Yes (but only Antoathecatae)
	Stylasteridae	Family	No	Yes	Yes	No	Yes	No	Yes (as subclass Hydroidolina)		Yes
	Zoantharia	Order	No	Yes	No	No	Yes	Yes	No		Yes
Echnidermata	Brisingida	Order	No	Yes	No	No	No	No	No		No
	Crinoida	Class	No	Yes	Yes (stalked only)	Yes (stalked only)	Yes (stalked only)	Yes	Yes		Yes (stalked only)
	Echinoidea	Class	No	No	No	No	Yes (as “Cidaroida”)	No	No		Yes (as "Cidaroida")
	Ophiuroidea	Class	No	No	No	No	Yes (as “Euryalida”)	Yes (as “Basket stars")	No		Yes (as "Euryalida")
Other Taxa	Arthropoda	Phylum	No	No	Yes (Chemosynthetic ecosystem decapods only)	No	Yes (Bathylasmatidae)	No	No		Yes (Bathylasmatidae)
	Ascidiacea	Class	No	No	No	Yes	Yes	Yes	No		Yes
	Bivalvia	Class	No	No	Yes (chemosynthetic communities)	No	Yes (as *Adamussium colbecki*)	No	Yes (chemosynthetic communities & subclass Gryphaeidae)		No
	Brachiopoda	Phylum	No	No	No	No	Yes	No	No		Yes
	Bryozoa	Phylum	No	Yes (as “all taxa within the orders Cheilostomatida & Ctenostomatida”)	Yes (only one species, *Eucratea loricata*)	Yes (Fenestrate taxa)	Yes	Yes	Yes (only Gymnolaemata & Stenolaemata)		Yes
	Polychaeta	Class	No	No	Yes (chemosynthetic communities)	No	Yes (as “Serpulidae”)	Yes (as “Serpulidae”)	Yes (chemosynthetic communities; infraclass Canalipalpata)		Yes (as "Serpulidae")
	Porifera	Phylum	No**	Yes (as Classes Demospongiae & Hexactinellida)	Yes	Yes	Yes (as Hexactinellida & Demospongiae)	Yes	Yes (class Demospongiae & subclasses Amphidiscophora & Hexasterophora within Hexactinellida)		Yes (as Hexactinellida & Demospongiae)
	Pterobranchia	Class	No	No	No	No	Yes	No	No		Yes
	Xenophyophoroidea	Suborder	No	No	Yes	Yes	Yes	No	No		Yes
Features	Chemosynthetic taxa	(multiple)	No	No	Yes (0–2,000 m)	No	Yes	No	Yes		Yes
	Seamounts as a whole	n.a.	No	No	Yes (VME element)	Yes	No	No	Yes (VME-indicator feature)		No
Management	Move-On Rule Use	n.a.	Yes but only for corals	Yes	Yes	Yes	Yes	Yes		Yes (no threshold though—encounter is enough)	Yes
	Other methods for identifying VMEs	n.a.	Fisheries drop camera surveys	Habitat suitability models for VME indicator taxa	Scientific advice (ICES), scientific surveys, multi-criteria assessments	Quantitative modelling, scientific advice	No	Commissioned scientific surveys		Scientific surveys	No

**Notes:**

NPFC, North Pacific Fisheries Commission; SPRFMO, South Pacific Regional Fisheries Management Organisation; NEAFC, Northeast Atlantic Fisheries Commission; NAFO ABNJ, North Atlantic Fisheries Organisation Areas Beyond National Jurisdiction; CCAMLR, Convention on the Conservation of Antarctic Marine Living Resources; SEAFO, Southeast Atlantic Fisheries Organisation; GFCM, General Fisheries Commission for the Mediterranean; SIOFA, Southern Indian Ocean Fisheries Agreement.

* During the review of this manuscript the taxonomy of Octocorallia and Alcyonacea were revised (McFadden, van Ofwegen & Quattrini, 2022). These revisions have not been incorporated by RFMOs yet and so the pre-revision taxonomy is retained here.

** During the revision of this manuscript, in December 2022 the NPFC began the process to approve select Porifera as VME indicators.

Another major caveat of this approach is that VME encounter thresholds used for ‘move-on rules’ vary among RFMO/As and are not always in place for all VME indicator taxa. While differences in thresholds between RFMO/As for some VME indicators can be expected because of regional differences in representative taxa and fishing gear configurations, thresholds would be expected to be similar for similar taxa and where similar gear is deployed. However, this situation may only rarely occur because of the coarse and varying levels of taxonomic aggregation that occurs for VME indicator taxa, which thereby can also include regional variation in the representative taxa. For example, the move-on threshold for the VME indicator “corals” in trawls ranges from 30 kg in NEAFC (NEAFC, 2014) to 60 kg for NAFO (NAFO, 2019) and the Southeast Atlantic Fisheries Organization (SEAFO) (SEAFO Conservation Measure 30/15, 2016), while South Pacific RFMO (SPRFMO) splits “corals” into multiple lower taxonomic groups with thresholds of 5–250 kg depending on the taxon (SPRFMO, 2019). Similarly for sponges, thresholds range from 50 kg for SPRFMO (SPRFMO, 2019) to 600 kg for SEAFO (SEAFO, 2016; SEAFO Conservation Measure 30/15, 2016). This threshold level variation raises the issue of the varying processes that RFMO/As use to define these thresholds, and the limited validation of those thresholds in terms of effectively detecting VMEs (which includes the role that catchability plays in this issue). For most RFMO/As, there are no specific encounter thresholds set for other VME indicators, such as xenophyophores, tube-dwelling anemones, or stalked crinoids (*e.g*. NEAFC, 2014). Furthermore, weight thresholds set for both corals and sponges may be too high for smaller, soft coral species such as sea pens and non-tetractinellid sponges which are of smaller size and biomass. Other RFMOs, such as NAFO and SPRFMO, have set thresholds for a wider range of VME indicators (NAFO, 2019; SPRFMO, 2019).

Despite these caveats, and often due to data limitations, most RFMO/As with the competence to manage bottom fisheries on the high seas continue to identify (and manage impacts on) VMEs in part using a move-on rule. However, as the ICES working group on deepwater ecology made clear “The damage caused by deep-sea bottom fishing activities to marine habitats and species, in particular VME indicators, is likely to remain unrecovered for decades to centuries. Reactionary management strategies such as the “encounter clauses” and “move-on rules” are of limited benefit to prevent significant adverse impacts because they still allow damage to occur which will gradually degrade ecosystems over time” (ICES, 2010).

Seafloor imagery data provides a potential additional or alternative means of identifying areas of VMEs on the seafloor. Seafloor imagery data from scientific surveys is becoming increasingly available for areas of the seafloor that harbor potential VMEs and is also a far less destructive method for mapping locations of, and gathering information on, VMEs. Imagery is playing a key role in improving our understanding of both the natural range of biomass of VME indicators per unit area, and of the proportion of that biomass that is captured in fisheries bycatch by trawls and longlines (*e.g*., Wassenberg, Dews & Cook, 2002). It can also be used for making predictive models for mapping the distribution of VMEs (*e.g*., Ross & Howell, 2013; Jackson et al., 2014; Rowden et al., 2017; Williams et al., 2020a; Morato et al., 2020). However, although imagery has informed some RFMO/A VME closures, there are currently no widely accepted quantitative criteria established to designate VMEs directly from images, and this approach instead has largely relied on “expert opinion”. Only a few recent articles have provided more quantitative guidance on establishing and using VME indicator density or biomass thresholds for identifying VMEs from imagery (*e.g*., Bullimore, Foster & Howell, 2013; Davies et al., 2015; Rowden et al., 2020; Williams et al., 2020a).

Thus, the goal of this study was to bring together an international working group of benthic ecologists from the Deep-Ocean Stewardship Initiative, VME Imagery Working Group, to establish first-pass consensus guidelines across geographic regions for designating VMEs from images. In this article four first-order questions are addressed: 1. Which taxa are considered VME indicators? 2. Can a VME be identified from a single image? 3. What criteria can we use to identify a VME from a single image? And 4. What are the thresholds (density or diversity) that need to be met to characterize a single image as a VME? Based on this work, we also make recommendations for management and highlight the next steps to be taken to continue to develop criteria for establishing VMEs from imagery.

**BOX 1—Definitions for VME-like designations and related regulatory frameworks**

The United Nations General Assembly (UNGA) adopted the term VME to refer to benthic ecosystems vulnerable to adverse impacts from bottom-contact fisheries. The UN FAO International Guidelines for the Management of Deep-Sea Fisheries in the High Seas, adopted in 2008, establish criteria that were subsequently incorporated into measures adopted by most of the regional fisheries management organizations with the legal competence to manage bottom fisheries on the high seas (DSCC 2016). Other international organizations and States have also developed related criteria or frameworks for identifying and/or designating sensitive deep-sea ecosystems. A brief, non-exhaustive list is given here, while some more specific criteria comparisons can be found in Gianni, Bos & Peijs (2012) and Ardron et al. (2014).

**Other international instruments that relate to definitions of VMEs**

Ecologically and Biologically Sensitive Areas (EBSAs)–Defined by the Conference of the Parties to the Convention on Biological Diversity in 2008 (COP 9) (Dunn et al., 2014), uses the Azores criteria (Uniqueness or Rarity; Special importance for life history stages of species; Importance for threatened, endangered or declining species and/or habitats; Vulnerability, Fragility, Sensitivity, or Slow recovery; Biological Productivity; Biological Diversity and Naturalness), to define larger areas that support the healthy functioning of oceans and their services (https://www.cbd.int/ebsa).

Areas of Particular Environmental Interest (APEIs)—One of several area-based tools used by the International Seabed Authority (ISA) to conserve representative or vulnerable communities and species at a regional scale (ISA, 2012; Wedding et al., 2013). More recently at regional workshops a spatial hierarchy of Areas and Sites in need of Protection or Precaution have also been developed (ISA, 2020). At a smaller scale, to enable improved knowledge of potential mining effects, are Impact Reference Zones (IRZs) and Preservation Reference Zones (PRZs) which should be comparable and support compare-contrast evaluations of disturbance effects in the IRZ (ISA, 2017).

Threatened and/or Declining Species and Habitats—The OSPAR Convention on the “Protection and Conservation of Ecosystems and Biological Diversity of the Maritime Area” uses the Texel/Faial criteria for species (Global importance; regional importance; rarity; sensitivity; key stone species and decline) and habitats (Global importance; regional importance; rarity; sensitivity; ecological significance and status of decline) and after that listed as Threatened and/or Declining Species and Habitats, by OSPAR (OSPAR Commission, 2019).

European Union/Commission (EU/EC)—The EU Deep-sea Access Regulation 2016/2336 requires managing bottom fisheries below 400 m depth to prevent SAIs on VMEs and bans bottom trawling in EU waters below 800 m (European Union, 2016).

Biotopes—Each VME could be thought of as a type of biotope (Connor, 1994; Olenin & Ducrtoy, 2006) With biotope describing a habitat of environmental conditions and the single community (biocenosis, *sensu* Dahl, 1908) that it hosts. Usually applied over a whole region, a seascape is classified into biotopes where one or more may be VMEs (Davies et al., 2015). OSPAR and the UK provide guidance that a biotope must cover a minimum area of 25 m^2^ (OSPAR, 2008a, 2008b; Parry, 2019).

**Example national efforts (regulation: terms, references)**

Canada—Oceans Act: Area of Interest (AOI), Marine Protected Areas (MPAs), Significant Benthic Areas (*e.g*., Ban et al., 2016, 2017, 2019a, 2019b, 2021).

Japan—Ministry of the Environment (2020a, 2020b): Natural Environment Conservation Area Designation and Conservation Plans.

New Zealand—Exclusive Economic Zone and Continental Shelf (Environmental Effects) Regulations 2013: sensitive environments, seamount closure areas, benthic protection areas (Brodie & Clark, 2003; Helson et al., 2010).

Norway—Regulations on fishing for the protection of vulnerable marine ecosystems FOR-2019-03-29-416: fishing regulation areas, including depth bans at 800 and 1,000 m. Regional management plans: particularly valuable and vulnerable areas (SVO; Eriksen et al., 2021).

Portugal—Portaria n° 114/2014: restricted bottom-trawling area, mandatory reporting and registration of sponge and coral bycatch. European Commission Council Regulation No. 1568/2005: Regulated fishing below 200 m in two areas in the Azores and the Madeira Canaries (European Commission, 2005).

South Africa—National Environmental Management: Protected Areas Act No 57, 2003: MPAs, Marine Living Resources Act: fisheries management areas, Critical Biodiversity Areas (CBAs) (Sink et al., 2019; Reed, Lombard & Sink, 2020; Harris et al., 2022).

United Kingdom—Marine and Coastal Access Act 2009: MPAs, MPA features (*e.g*., Henry & Roberts, 2014a, 2014b).

United States—Magnuson-Stevens Fisheries Conservation and Management Act (MSA): essential fish habitat (EFH), deep-sea coral discretionary authority (NOAA, 2010; Hourigan, 2015; Hourigan, Etnoyer & Cairns, 2017).

**Certifications**

The Marine Stewardship Council (MSC) includes the FAO VME definition in the determination of sustainable fisheries (MSC Fisheries Standard and Guidance v2.01, 2018).

### Survey methodology

At the 15^th^ Deep-Sea Biology Symposium in 2018 in Monterey, California, multiple presentations based on image data, as well as discussions among a subset of the attendees, indicated there was a critical need for consensus criteria for identifying VMEs from imagery data. A working group was formed under the auspices of the Deep-Ocean Stewardship Initiative (DOSI) to focus on developing these criteria. To reduce their carbon footprint, the working group met remotely during 2019–2021 and collated global datasets to discuss their expert opinions on what they considered to be a VME. Discussions covered a comparison of VME indicators among regions, how presence of a VME can be recognized in a single image, the number of images needed to determine whether a site is a VME, and areal extent and thresholds. Individual scientists shared images of the seafloor in areas they were working and discussed attributes of the images that led them to the conclusion that an image did or did not show a VME (details below). Incorporating opinions from deep-sea experts and managers from 15 countries and images from around the globe, this study represents the consensus of the discussions undertaken and reviews the remaining open questions and challenges for using imagery to determine whether sites are VMEs.

## Results and Discussion

### Question 1: Which taxa are considered VME indicators?

First, a list of taxa already designated as VME indicators by RFMO/As was compiled. VME indicators are defined as those taxa that meet at least one of the FAO criteria (uniqueness or rarity, functional significance, fragility, life history traits that contribute to slow recovery, or areas of structural complexity-see Table 1 for more detail) and are therefore proxies for the possible presence of a VME (UNGA Resolution 64/72, 2009; FAO, 2009). It is important to recognize here that a VME is an ecosystem, not simply the taxa that provide habitat structure, and that using VME indicators as a proxy to determine areas to protect as VMEs provides a mechanism for protecting biodiversity overall.

The list of VME indicators currently identified by RFMO/As indicates that what is considered a VME indicator taxon varies considerably by region (Table 2). The cnidarian orders Alcyonacea, Scleractinia, and Antipatharia are the only taxa with members that are considered VME indicators across all RFMO/A regions. Additionally, many RFMO/As only include subsets of a given taxon, for example, a specific family or order within a class, while other RFMO/As include the whole class. For example, within the order Alcyonacea, there is some disparity on which taxa are included. Some RFMO/As (*e.g*., General Fisheries Commission for the Mediterranean (GCFM) and Convention on the Conservation of Antarctic Marine Living Resources (CCAMLR)) include all Alcyonacea, while NAFO only includes the alcyonaceans formerly known as Gorgonacea. Similarly, within the Scleractinia, the EU recognizes cup corals as VME indicators whereas other RFMO/As do not (*e.g*., NAFO; ICES, 2020).

In some cases, RFMO/As omit a taxon from a region because that VME indicator taxon has not been found in that region. While it is understandable that VME indicators are drawn from inventories of known taxa, large portions of many RFMO/A convention areas are unexplored, and further surveys may reveal missing taxa that are considered VME indicators in other areas. An example of this was the discovery of deep-sea scleractinian reefs on seamounts in the North Pacific (Baco et al., 2017), after over two decades of exploration in the region and speculation that seawater chemistry in the region would prevent reef formation (Guinotte et al., 2006). In other cases, taxa are present and known to be structure forming in that region and yet are not included as VME indicators, even though they should be based on the FAO criteria (Table 1). Examples include sponges, xenophyophores, bryozoans, and chemosynthetic ecosystem taxa.

Sponges are very abundant in the North Pacific (*e.g*., Krautter et al., 2001; Campbell et al., 2009; Parrish et al., 2015; Kennedy et al., 2019; Downey, Fuchs & Janussen, 2020), occur within heavily fished areas (Du Preez, Swan & Curtis, 2020), can be caught in the North Pacific Fisheries Commision (NPFC) convention area by bottom trawls with moderate frequency (Miyamoto & Kiyota, 2017), and at the time of submission of this manuscript are listed as VME indicators in every single RFMO/A region except the NPFC. This discrepancy has been identified by global experts (FAO, 2019) and contradicts the known ecological importance of sponges within the NPFC region (*e.g*., Convention on Biological Diversity (CBD), 2016) and the inclusion of sponges in the management plans of surrounding EEZ MPAs (Marine Conservation Institute, 2021).

Among the more unusual VME indicators are giant, sediment-agglutinating protozoans called xenophyophores. Recognized as VME indicators by NEAFC, NAFO, and CCAMLR, these large foraminifera can attain high densities (>1/m^2^) on hard and soft substrates on seamounts, continental margins, and abyssal plains (Levin & Thomas, 1988; Gooday, Aranda da Silva & Pawlowski, 2011; Amon et al., 2016) and are known to host snailfish embryos (Levin & Rouse, 2019) and diverse assemblages of invertebrates (Levin et al., 1986; Levin, 1991). They are extremely fragile and vulnerable to disturbance, dominate in the CCZ area targeted for polymetallic nodule mining (Gooday et al., 2017; Gooday, Durden & Smith, 2020), and occur within fishing depths (*e.g*., Levin et al., 1986). Thus, they meet several of the FAO criteria, but are not yet listed by most RFMO/As, despite being found globally (Ashford, Davies & Jones, 2014).

Bryozoans are another taxon that meet multiple FAO criteria, and while included by SPRFMO, NEAFC, and CCAMLR, they are overlooked by NPFC, and only one species (*Eucatea loricata*) is currently considered as a VME indicator by NAFO. Habitat-forming bryozoans can provide habitat for diverse assemblages at the centimeter-to-meter scale, with associated assemblages comprised of more than 130 non-bryozoan species including Mollusca, Annelida, Arthropoda, Cnidaria, Porifera, and Echinodermata (Schlacher et al., 2010; Wood et al., 2012; Lombardi, Taylor & Cocito, 2020). Biogenic structures formed by bryozoans can attain significant sizes (up to several meters high) and can extend over 1,000 km (Wood et al., 2012; Lombardi, Taylor & Cocito, 2020). They are common in the Southern Ocean, New Zealand, Australia, the North Pacific around Japan, the northern Mediterranean, Bahamas, North-East Atlantic, and the North Sea (Lombardi, Taylor & Cocito, 2020).

Similarly, structure-forming chemosynthetic ecosystem taxa (*e.g*., mussels, tubeworms, clams) are not recognized as VME indicators in all regions in which they are known to occur, *e.g*., SPRFMO currently does not recognize them despite the known presence of hydrothermal vents on the East Pacific Rise and in ABNJ sections of the Kermadec Ridge (reference map in Menini & Van Dover (2019)). (However, bottom trawling by SPRFMO nations does not currently occur in these areas of the high seas).

Further issues with VME indicator lists used for move-on rules result from them being based on fauna caught as trawl or longline bycatch, which may result in exclusion of the many smaller or more fragile taxa that are destroyed or washed out of trawls (Wassenberg, Dews & Cook, 2002; Heifetz, Stone & Shotwell, 2009; Jones & Lockhart, 2011; Pitcher, Williams & Georgeson, 2019). Ecosystems are typically considered on the scale of epibenthic megafauna, particularly those with well-understood life histories, while smaller scales and poorly understood taxa are currently overlooked. Infauna, all size classes of which include ecosystem engineers, are also not considered. Furthermore, most RFMO/As list higher taxonomic levels to simplify taxonomic identification for fishers or fisheries observers, which can result in both inclusion and exclusion of taxa unintentionally.

A final issue is geographic bias in contributions to VME assessments. Seafloor imagery being used to augment fishery-based designation of VMEs is more common in some regions such as the North Atlantic or NE Pacific, and extremely limited in much of the ABNJ in the Indian Ocean, South Pacific and South Atlantic (Howell et al., 2020). Additionally, not all ocean regions have RFMO/As with competence to manage seafloor impacts of fishing. The tropical East Pacific, SW Atlantic, tropical North Atlantic, the NE Indian Ocean (including the Bay of Bengal) are examples of large international seabed areas without such RFMO/As in place (Bell, Guijarro-Garcia & Kenny, 2019), and thus have not undertaken VME designation. Having a consistent set of VME indicators across regions could help to simplify the designation process in all areas, especially those where RFMO/A programs are still developing. Beyond fisheries regulation, having such a consensus could benefit spatial management efforts in other sectors, including regulation of deep seabed mining, and oil and gas exploration. Consensus lists could also broadly and proactively inform biodiversity conservation efforts in ABNJ.

Thus, based on these observations and caveats, the first recommendation is that there is a need to establish a consensus list of the key VME indicators across regions that is continually updated when new taxa and communities are encountered, or more scientific information is gained that allows new assessments against the FAO criteria, and that benefits from observations made using imagery.

### Question 2: Can a VME be identified from a single frame?

**Figure 2:**
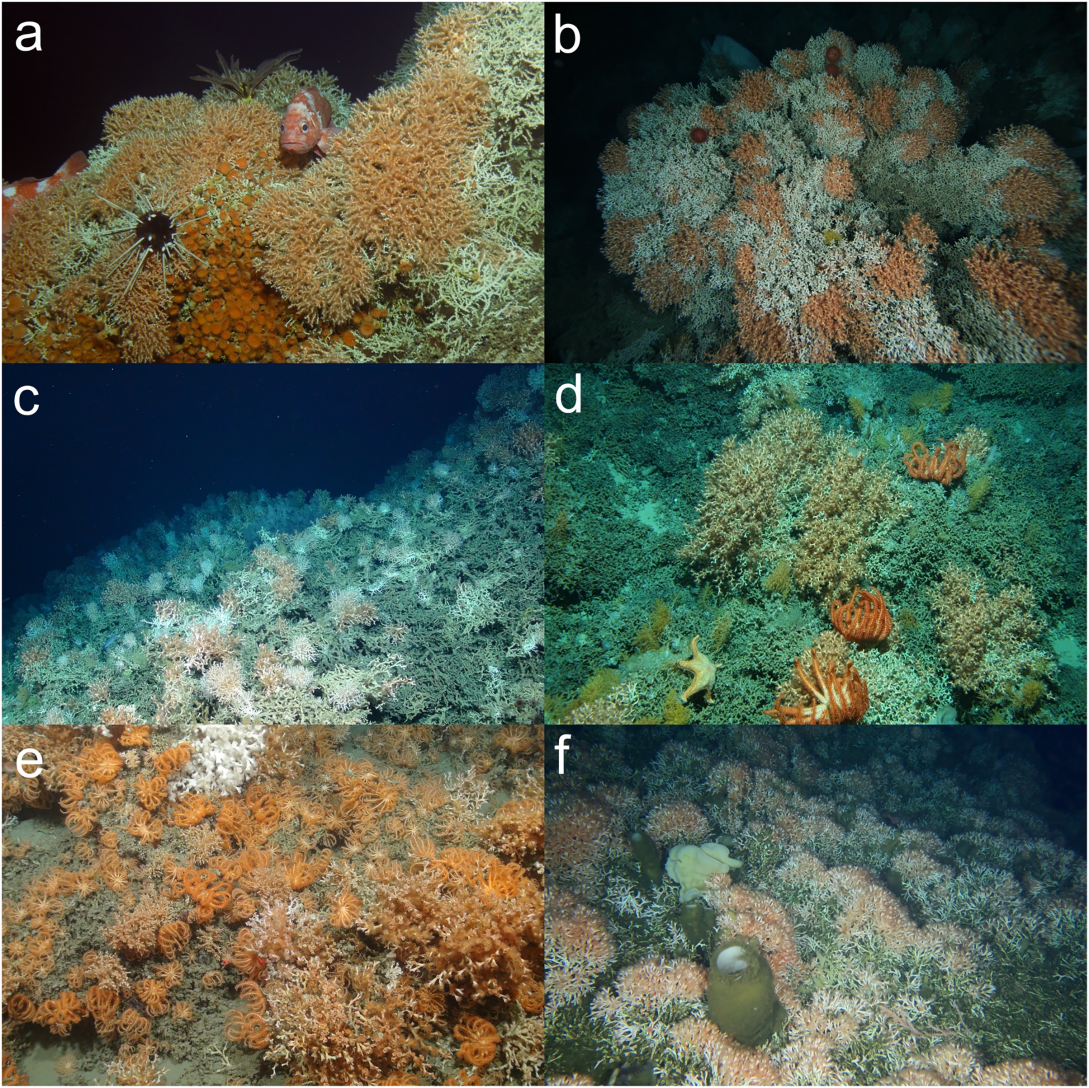
Examples of deep-sea scleractinian reefs that can be identified as a VME from a single image. (A) *Solenosmilia variabilis* Duncan, 1873 reef with associated rockfish and invertebrates on Colahan Seamount on the Northwestern Hawaiian Ridge (Baco, Morgan & Roark, 2020, CC BY-NC-ND 4.0). (B) A thicket of the reef-forming stony coral *Solenosmilia variabilis* at 1,140 m depth on seamount z16, Southern Tasmania, Australia. Image: CSIRO, Survey SS200611. (C) A *Desmophyllum pertusum* (formerly *Lophelia pertusa* (Linnaeus, 1758)) reef on Anton Dohrn Seamount west of Scotland. Image: NERC funded Deep Links Project-Plymouth University, Oxford University, BGS, JNCC. (D) A thicket of the reef-forming stony coral *Solenosmilia variabilis* at 1,000 m depth on the summit of a small seamount off New Zealand; brisingid seastars, small crinoids, and fluffy octocorals are also present. Image: NIWA. (E) Cold-water corals *Desmophyllum pertusum* and *Madrepora oculata* Linnaeus, 1758, with brisingidae within Explorer Canyon, North East Atlantic. Image: JC125 cruise, National Oceanography Centre, Southampton. (F) A *Desmophyllum pertusum* reef at ~200 m in the fjords of the Central Coast of British Columbia, Canada. Image: Fisheries and Oceans Canada. © His Majesty the King in Right of Canada, 2023.

**Figure 3:**
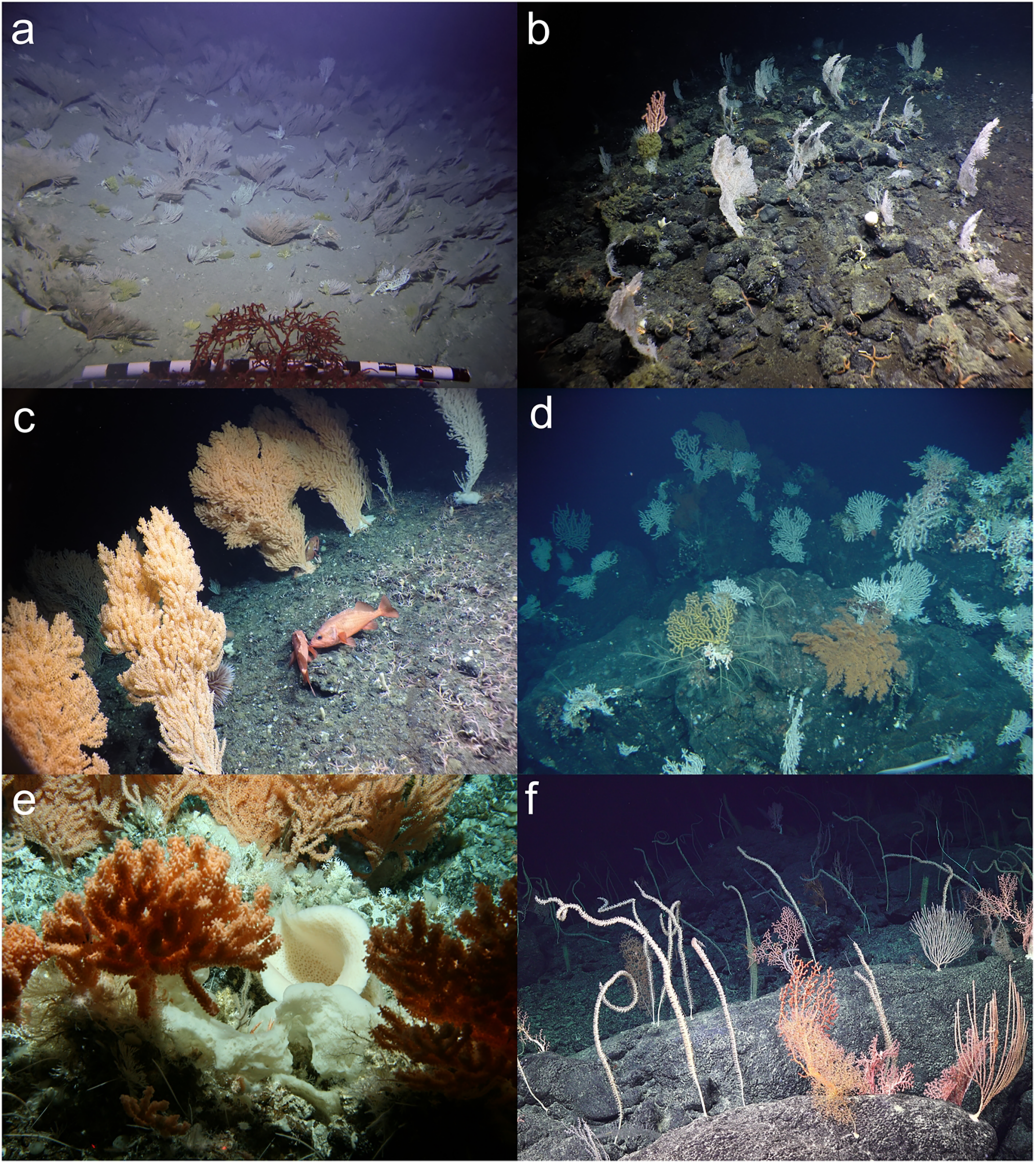
Examples of coral gardens that can be identified as a VME from a single image. (A) An octocoral and antipatharian garden on Koko Seamount in the Emperor Seamount Chain (Baco, Morgan & Roark, 2020, CC BY-NC-ND 4.0). (B) Extensive *Parastenella* spp. octocoral gardens encircle the slopes of the Dellwood Seamounts, in the Canadian Northeast Pacific. Image: Ocean Exploration Trust/Northeast Pacific Seamount Expedition Partners, D. Fornari (WHOI-MISO Facility). © His Majesty the King in Right of Canada, 2023. (C) A forest of red and white tree corals (dominated by *Primnoa pacifica* Kinoshita, 1907) on the plateau break of SGaan Kinghlas-Bowie Seamount (~600 m depth), one of the tallest seamounts in the Northeast Pacific and Canada’s shallowest underwater volcano. Also visible are some of the rougheye rockfish (*Sebastes aleutianus* (Jordan & Evermann, 1898)) hiding between the 1–2 m stands. Image: Ocean Exploration Trust/Northeast Pacific Seamount Expedition Partners, D. Fornari (WHOI-MISO Facility). © His Majesty the King in Right of Canada, 2023. (D) Cold-water coral garden within the Menez Gwen protected area at the Azores Marine Park. Image: Missão *Seahma*, 2002 (FCT, Portugal *PDCTM* 1999MAR15281). (E) A mixed VME of primnoid corals and sponges on a seamount south of New Zealand on the Macquarie Ridge. Image: NIWA. (F) An octocoral garden on O’Brian Seamount in the North Pacific. Image: A. Baco FSU, E.B. Roark and K. Shamberger TAMU, NSF, and the ROV JASON II.

**Figure 4:**
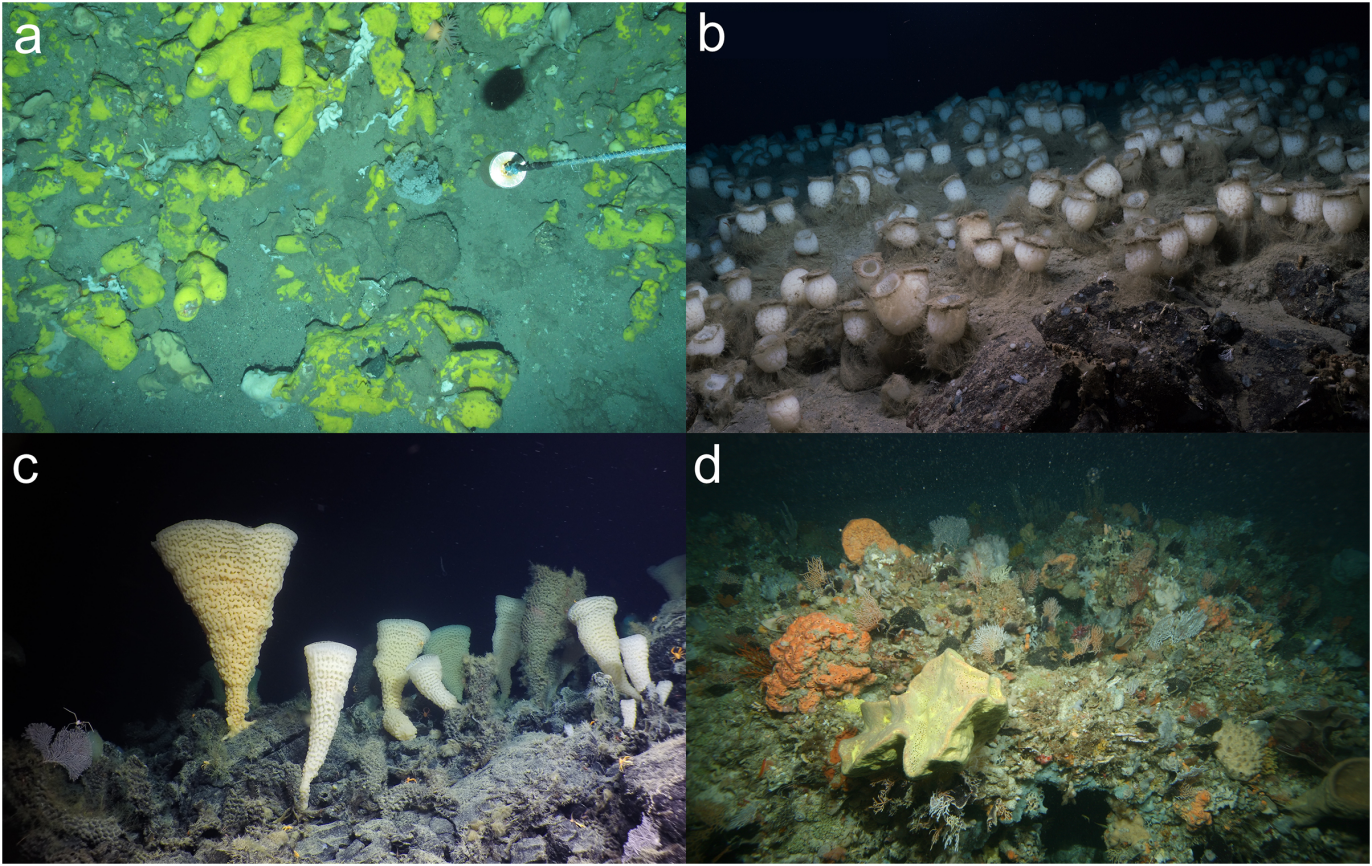
Example images of sponge aggregations that were considered a VME from a single image. (A) A deep-sea sponge aggregation, comprising *Geodia* sp., from the Faroe Shetland Channel in UK waters. Image: JNCC and Marine Scotland Science survey 1517S. (B) Sponge ground, formed by the bird’s nest sponge *Pheronema carpenteri* (Thomson, 1869), at the flank of Pico Island in the Azores (800 m). Image: Rebikoff Foundation. (C) A city of glass sponges covers the summit of Explorer Seamount, a supervolcano in the Northeast Pacific and Canada’s largest underwater volcano. This new species of *Pinulasma* dominates otherwise relatively bare and exposed lava at 800 m depth, adding vertical relief and complex structure to the terrain. Image: Ocean Exploration Trust/Northeast Pacific Seamount Expedition Partners, D. Fornari (WHOI-MISO Facility). © His Majesty the King in Right of Canada, 2023. (D) Sponge and bryozoa/hydroid community at 85 m depth off Jurien Bay, Western Australia. Image: CSIRO, ‘Voyage of Discovery’ Survey SS200507.

**Figure 5:**
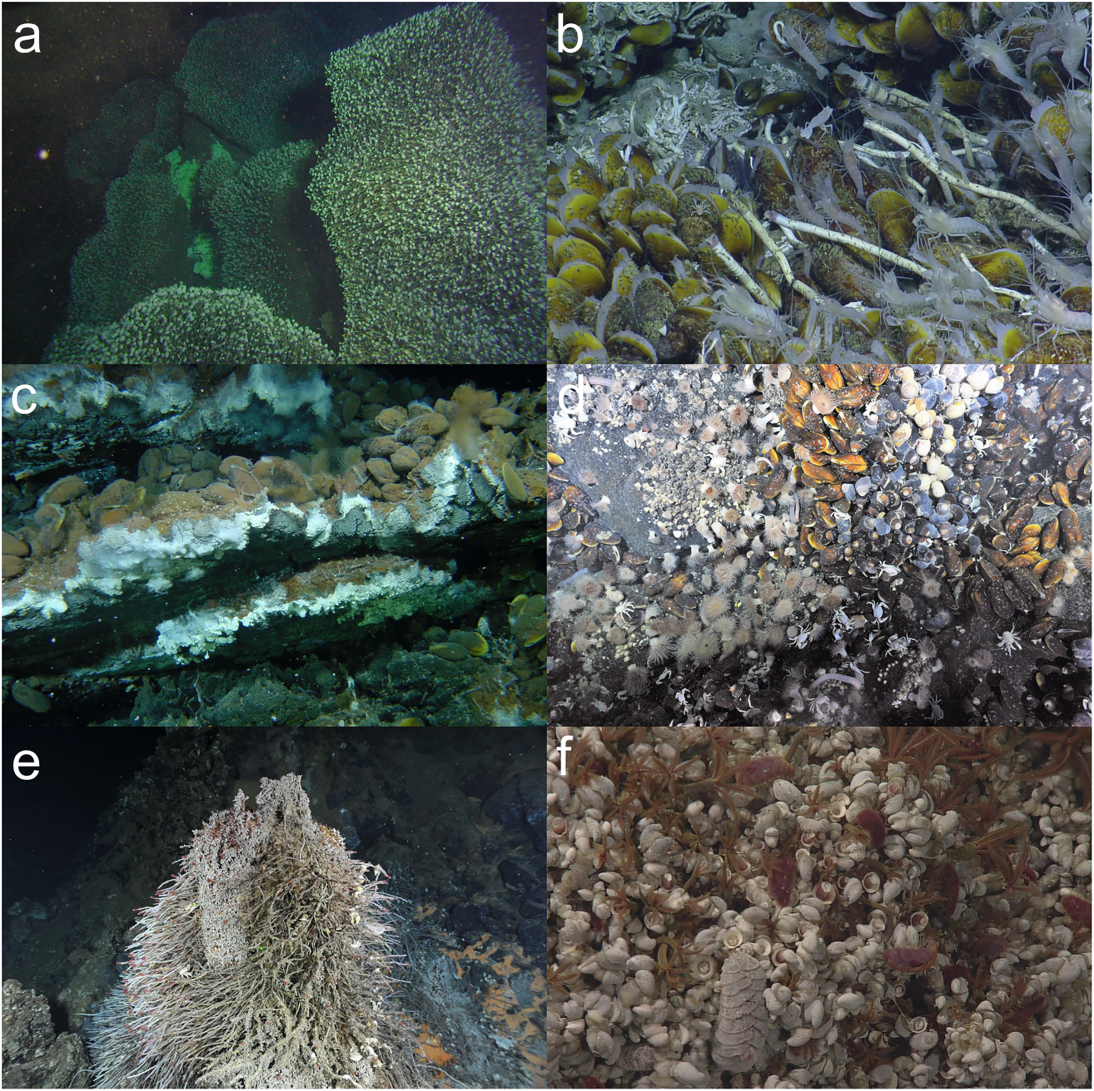
Example images of chemosynthetic ecosystems that can be identified as a VME from a single image. (A) Stalked barnacles completely covering rocks near a hydrothermal vent on the Kermadec Volcanic Arc north of New Zealand. Image: NOAA, NIWA, GNS. (B) Methane seep mussel and tubeworm community with associated epifauna near Trinidad and Tobago. Image: Ocean Exploration Trust, EV Nautilus cruise NA054. (C) Hydrothermal vent chimney with the endemic vent mussel *Bathymodiolus azoricus* von Cosel, Comtet & Krylova, 1999 (Threatened species; Thomas et al., 2021) at the Lucky Strike protected area at the Azores Marine Park. Image: © Missão *Seahma*, 2002 (FCT, Portugal *PDCTM* 1999MAR15281). (D) Hydrothermal vent covered in a chemosynthetic community of provannid snails *Alviniconcha* spp. and *Ifremeria nautilei*, and the mussel *Bathymodiolus septemdierum* Hashimoto & Okutani, 1994 with associated invertebrates from the Lau Basin hydrothermal vents, in the Kingdom of Tonga (Threatened species; Thomas et al., 2021). Image: SOI, ROPOS, Du Preez. (E) A low flow hydrothermal vent chimney covered in chemosynthetic white bacterial mats and clumps of endosymbiont containing tubeworms (*Ridgeia piscesae* Jones, 1985) from Endeavour Hydrothermal Vent MPA, Canada. Image: Fisheries and Oceans Canada. © His Majesty the King in Right of Canada, 2023. (F) Zoomed-in image of a clump of sulfide worms (*Paralvinella sulfincola* Desbruyères & Laubier, 1993)—a pioneer species that facilitates colonization by the limpets (*Lepetodrilus fucensis* J. H. McLean, 1988) and other vent associated animals. High flow site at Endeavour Hydrothermal Vent MPA, Canada. Image: Fisheries and Oceans Canada. © His Majesty the King in Right of Canada, 2023.

**Figure 6:**
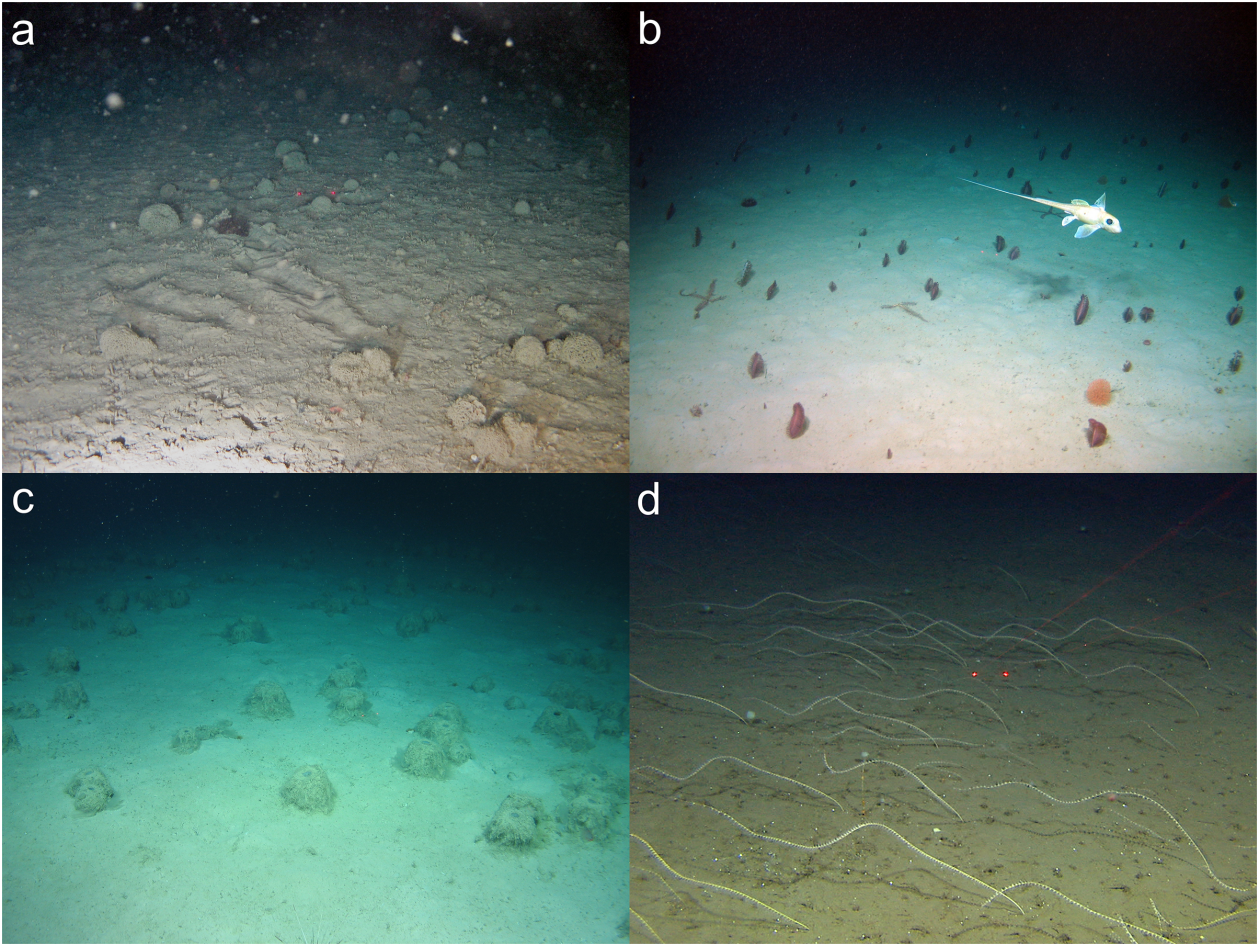
Examples of soft-sediment communities that can be considered VMEs from a single image. (A) A *Syringammina fragilissima* Brady, 1883 xenophyophore aggregation at the Darwin Mounds Marine Protected Area northwest of Scotland. Image: National Oceanography Centre, UK (B) mixed sea pens and an *Acanella arbuscula* bamboo coral on the continental slope west of Ireland. Image: the SeaRover project, co-funded by the Irish Government and the European Maritime and Fisheries Fund 2014–2020. (C) *Pheronema carpenterii* Thomson, 1869 sponge aggregation in the Porcupine Seabight southwest of Ireland. Image: University of Plymouth, Marine Institute Ireland, Eurofleets 2. (D) *Radicipes gracilis* meadow at 667 m near Bear Island, Norway. Image: Mareano programme, Institute of Marine Research, Norway, cruise 2009105.

**Figure 7:**
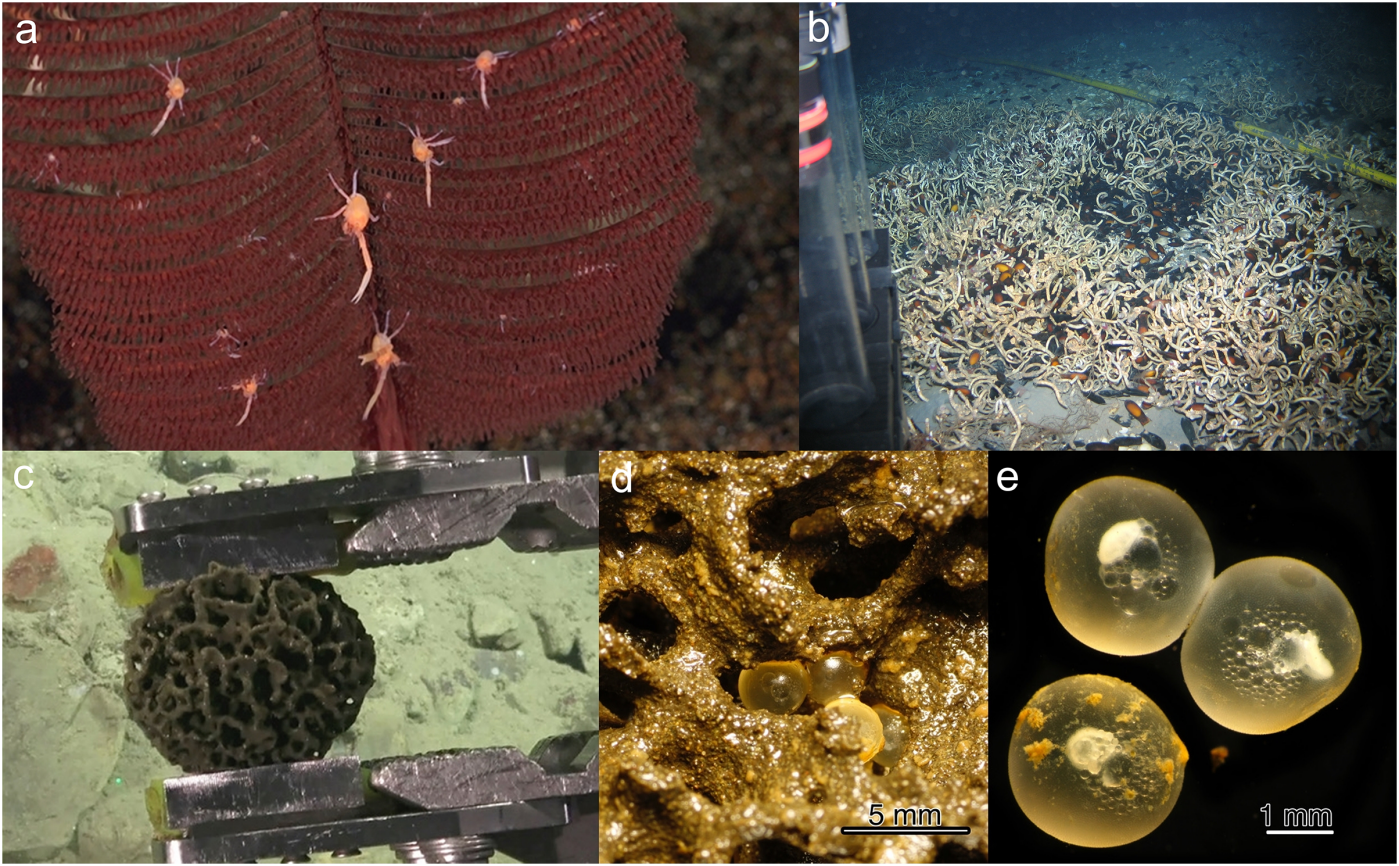
Examples of VME Indicators acting as nurseries. (A) *Bathypathes* (black coral) as nursery habitat for juvenile galatheid crabs. Costa Rica margin Las Gemmelas seamount. Image: RV Falkor/SuBastian FK190106 Dive S0225; Schmidt Ocean Institute, CC-BY-NC-SA 3.0. (B) Egg capsules of the deep-water catshark *Galeus melastomus* Rafinesque, 1810 in a tubeworm field (*Lamellibrachia* spp.) at the North Alex Mud Volcano, eastern Mediterranean Sea. Image: T. Treude (C) Xenophyophore on the Costa Rica Margin. Image: Schmidt Ocean Institute. (D) Fish eggs attached to *Reticulammina* sp. test, identified as *Paraliparis* sp. *via* DNA analysis (GenBank MN509401); eggs were dead upon discovery, after having been in shipboard incubation experiments for 10 days. (E) Closer view of fish eggs from (D). Image for (D and E): Levin & Rouse (2019). Photographs by Greg Rouse.

**Figure 8:**
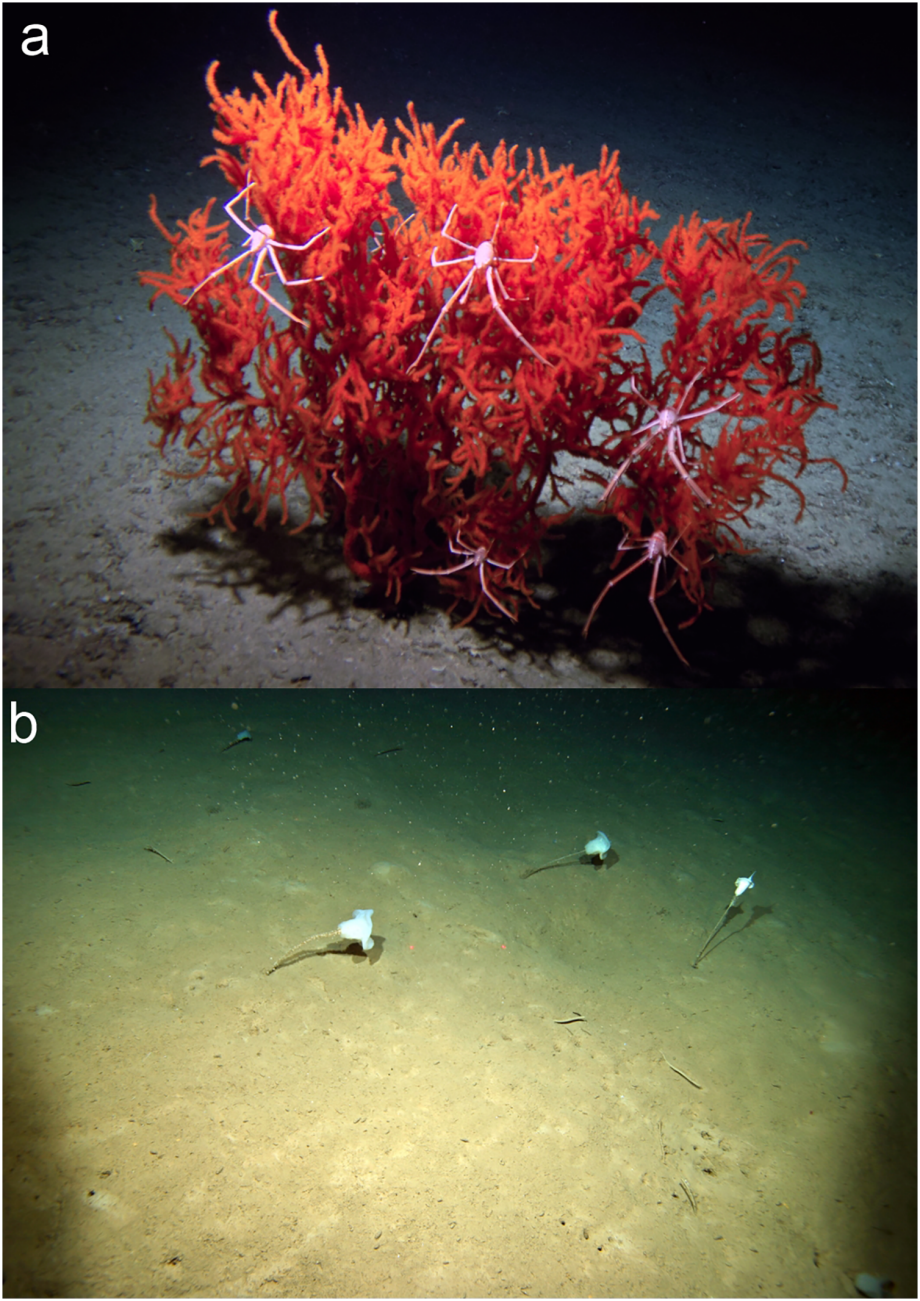
Example images of VMEs that were harder to distinguish from a single image. (A) A large antipatharian with associated galathaeid crabs from the Northwestern Hawaiian Ridge. Image: A. Baco FSU, E.B. Roark TAMU, NSF, with HURL Pilots T. Kerby and M. Cremer. (B) Low-density fields of stalked glass sponges (genus *Hyalonema*) at 650 m depth, extending over ~80% of the 2 km long video transect off Ningaloo Western Australia. Image: CSIRO, ‘Voyage of Discovery’ Survey SS200507.

In some RFMO/As (*e.g*., the NPFC), VME imaging surveys to inform fisheries are conducted with drop cameras, which may only provide a single image per lowering. Additionally, even in multi-image and video surveys, single images or single frames from video are often the sampling unit, and therefore the starting point for analyses (*e.g*., Rowden et al., 2017; Williams et al., 2020a). Thus, the next question to address was: can a VME be identified from a single image or single video frame? Even without a consensus on which taxa are VME indicators across regions, to resolve this question we used the list of VME indicators in at least one region (Table 2) as a starting point, in concert with the FAO criteria (Table 1). For this task, scientists from different regions each shared 3–5 images that they considered to be a VME, to determine whether others agreed with this assessment. From this qualitative exercise, it was concluded that in some cases, all could agree that a single image showed a VME. Examples of single image VMEs are included in Figs. 2–8. Common themes to the agreed frames were the presence of reef or of an octocoral or antipatharian garden (Figs. 2 and 3). It is known that both live and dead corals can be important habitat (Mortensen & Fosså, 2006) and that deep-sea scleractinian reefs are fragile and comprised of species with life history characteristics that make them vulnerable (reviewed in Clark et al., 2016; Rogers et al., 2018). For example, Fig. 2A depicts a well–developed scleractinian reef, that has a commercial fish sitting on it, while several other invertebrate species can be seen in the image, all in clear association with the coral structure. This image meets all the FAO criteria, and the group consensus was that this is a VME.

Other examples where there was agreement included:

- Figure 3A, an image of an octocoral and antipatharian garden, with high density of individuals, a diversity of species, and encompassing a relatively large area within the field of view.
- Sponge reefs and gardens (Figs. 4, 6C).
- A well-developed chemosynthetic ecosystem (Fig. 5).
- A high density of a single VME indicator (Figs. 2, 3B, 3C, 4A, 4B, 5A–5C, 6).
- Multiple VME indicators in the same image (Figs. 3A, 3D–3F, 4D, 5B, 5D–5F).
- Images that demonstrated an association of other megafauna with the VME indicator(s) (Figs. 2 A, 2D–2F, 3C, 5B and 5F).
- Visible evidence of spawning or use of the VME indicator(s) as a nursery habitat (Figs. 7A and 7B).

Assessments were not always this simple, however. For example, in the image in Fig. 8A, there is an antipatharian coral that is clearly acting as a habitat for other taxa, but it is just one large coral colony. The question of whether this image was sufficient to represent a VME generated considerable debate. Similarly, assigning an image or video frame of four *Hyalonema* sponges as a VME was debatable, without the contextual knowledge of this frame being part of a continual patch of these sponges (Fig. 8B). It is noted that larger fields of view at an oblique angle to the seafloor can help imply this context within a single image (*e.g*., Figs. 6A–6C).

### Question 3: What criteria can we use to identify a VME from a single image?

**Figure 9:**
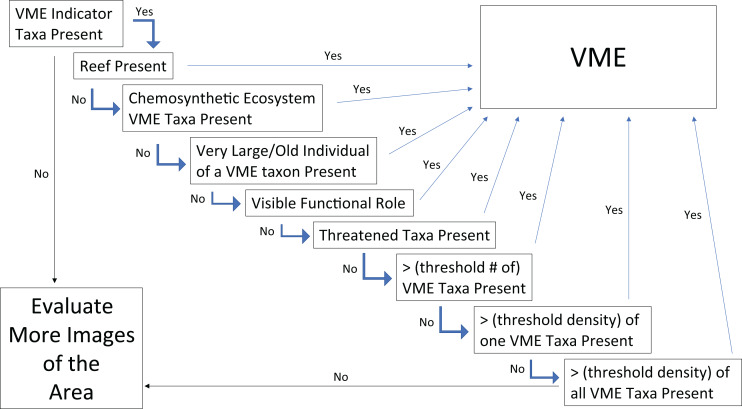
Flow chart for determining whether the faunal community in a single frame or image represents a VME. If a “Yes” is obtained in any step, the image can be considered a VME and no further steps need to be tested. A more in–depth explanation of each box along with explanations of the associated FAO criteria can be found in the text under Question 3.

Given the consensus that certain VMEs could be discerned from a single image, the next step was to assess which qualities led to the conclusion that it was an image of a VME? And relatedly, which of the FAO criteria (Table 1) can be captured in a single image to help make the determination? The images with agreed VME presence were reviewed to assess which aspects of the image contribute to this conclusion. Building on the points of consensus outlined in Question 2, a simple flow chart was constructed for designating VMEs from single images (Fig. 9). The first step in this chart is to assess whether there are VME indicators present, if not then the conclusion is that the image does not have evidence of a VME and more images from the site need to be evaluated. If VME indicators are present, the FAO guidelines state that “merely detecting the presence of an element itself is not sufficient to identify a VME” (FAO, 2009). Thus, the first step of the flow chart is the “element detection” step, and the second step and all subsequent steps introduce additional factors and decisions that aid in identifying a VME.

From this point, if any of the given steps lead to a “Yes”, then the image can be considered to represent a VME and no further steps need to be tested. For example, the next step is answering the question, “is there reef present”? If the answer is Yes, then the area in the image can be considered a VME. This can be a scleractinian reef or a sponge reef, as reefs meet most or all of the FAO criteria in Table 1 (see Question 2 above). The reef can also be alive or dead, because dead coral reef has been shown to host as much or more diversity as live reef in some areas (Mortensen & Fosså, 2006). Similarly, skeletal remains of sponges (dead reef, spicule mats, and body stalks) have been shown to provide suitable substrate for settlement of sponge juveniles and other benthic epifaunal or infaunal organisms, increasing local diversity (Bett & Rice, 1992; Beaulieu, 2001; Dunham et al., 2018).

If there is no reef present, then the next question to evaluate is, are there chemosynthetic ecosystem taxa present? What are considered VME indicators for chemosynthetic ecosystems varies regionally, some include endemic taxa, some specifically list bivalves, others decapods, and still others specifically list polychaetes (Table 2); however, any taxa that create structure would count. Like reefs, chemosynthetic ecosystems meet many of the FAO criteria including Structural Complexity—tubeworms and molluscs often harbor significant epifauna (Van Dover & Trask, 2000; Van Dover, 2002; Van Dover et al., 2003; Guillon et al., 2017; Gollner et al., 2021); Functional Significance—both vents and seeps have been observed to act as nurseries for chemosynthetic and non-chemosynthetic taxa (Gollner et al., 2021; Salinas-de-León et al., 2018; Sen et al., 2019; Turner et al., 2020; Fig. 7B); Uniqueness or Rarity, as they generally occur in discrete areas and exhibit regional endemism of fauna (Gollner et al., 2021); Fragility and Life History characters–at seeps, tubeworms have been documented to live for 200–800 years, fitting with the characteristics of being slow-growing and long-lived (Durkin, Fisher & Cordes, 2017).

If neither of the previous two conditions are met, the next step of Large/Old Individuals may capture one or several of the FAO criteria of: Uniqueness or Rarity, Functional Significance, and Fragility, and came from a discussion of size. More individuals of a smaller size fit into a given space than of larger individuals, hence single individuals alone might not meet other criteria in the flow chart. However, the large/old individuals may be unique or rare for the given location. From a functional perspective, large individuals may contribute disproportionately to reproductive success (*e.g*., Beiring & Lasker, 2000; Fountain, Waller & Auster, 2019; Beazley & Kenchington, 2012). It is also often the case that large individuals provide a habitat for many associated fauna (Buhl-Mortensen & Mortensen, 2004; Wagner, Luck & Toonen, 2012). In terms of fragility, it has been shown that most (or many) deep-sea corals have maximum longevities of tens to hundreds of years, and some corals may live for over 1,000 to 4,000 years (Roark et al., 2006, 2009; Prouty et al., 2017), which would make recovery impossible on a 5–20 year timescale, (which is the time frame for recovery established in the FAO Guidelines (paragraph 19)). Relatedly, if trawled, large individuals might make a significant contribution to meeting the weight thresholds for fisheries move-on rules. Thus, large or old individuals in an image should also result in a VME designation. As ‘large’ is a relative concept we suggest a benchmark of an individual (or discrete colony) of sufficient size to make it likely to be more than 100 years old. We note that in many cases, determination of what constitutes an “old” individual for a species will require previous information.

If none of these previous criteria are met, but there is a visible functional role, *e.g*., as a nursery, then the image can also be considered a VME (Fig. 7). The FAO criteria (Table 1) refer to VMEs as “areas or habitats” necessary for the survival, function, reproduction, or recovery of fish stocks…”. It has been documented for several species of corals and sponges that, for example, elasmobranchs and other commercially important species may attach or hide their eggs in them (*e.g*., Etnoyer & Warrenchuk, 2007; Busby et al., 2012; Baillon et al., 2012). Other examples include the *Bathyraja* deep-sea skate spawning ground described on Shiribeshi Seamount (Hunt, Lindsay & Shahalemi, 2011), which led to the designation of this seamount as an MPA, and the *Muusoctopus* octopus spawning site off Costa Rica (Hartwell, Voight & Wheat, 2018), and more recently on Davidson Seamount off California (King & Brown, 2019). Similarly, if juvenile or newly recruited fishes or other taxa are present in a site (Fig. 7), it could warrant VME designation. However, it should be noted that in many cases the nursery role of VME indicators is not apparent from survey images and requires finer-scale examination of specimens (*e.g*., Figs. 7C–7E; Baillon et al., 2012).

Also related to the Functional Significance criterion, and the Uniqueness and Rarity criterion, for the flow chart box of “Threatened Taxa”, the FAO Guidelines in paragraph 42 (Table 1) refer to VMEs as “areas or habitats … necessary for the survival, function, … reproduction, or recovery of … rare, threatened, endangered or endemic species.” “In the case of confirmed or likely rare or endemic species the presence of these species should be sufficient grounds to identify the area as a VME.” This guideline implies that areas where rare or endemic species have been found or are likely to occur should be designated as VMEs, irrespective of whether biogenic habitat or listed VME indicators are present. Many hydrothermal vent molluscs would fit into this decision criterion, with 72% of all vent mollusc species globally, listed as critically endangered, endangered, or threatened on the IUCN Red List (Thomas et al., 2021). Many of these species are not themselves listed as VME indicators, but instead occur as epifauna on the other larger vent taxa and chimney surfaces. Observations of these listed species would warrant a VME designation of an area regardless of the presence of other VME indicators.

Of course, the VME indicators themselves may be rare or threatened, *e.g*., the octocoral *Isidella elongata* Esper, 1788 is on the IUCN Global Red List as critically endangered along with nine other deep-sea coral species that are categorized as endangered and seven that are listed as vulnerable (IUCN, 2016). Thus, regardless of whether the threatened species is a VME indicator or not, or whether other VME indicators are present or not, according to the FAO criteria, the presence of a threatened species is enough to designate a VME area.

If none of the previous conditions are met, the last three flowchart criteria focus on the diversity and density of taxa in an image. The first criterion is whether there is a threshold number of VME indicators present in the image. The next applies to monotypic stands of VME indicators that meet a minimum density threshold. And the last step looks at the density of all taxa together to allow for the fact that there may be one or two individuals of one VME indicator and one or two of another, so the image would not meet either of the previous criteria, but combining them together can still meet the FAO criteria. The final step of the flow chart is that if the image meets none of these criteria, then that single image does not depict a VME. To be certain about the implied absence of VMEs in that area though, more images should be evaluated.

### Question 4: What are the thresholds (density or diversity) that need to be met to characterize a single image as a VME?

The last three steps of the flow chart each include a placeholder “threshold” value. Within the FAO criteria, the definition of “Structural Complexity” for designating a VME is given as: “v. Structural Complexity–an ecosystem that is characterized by complex physical structures created by significant concentrations of biotic and abiotic features” (FAO, 2009). The term “significant concentrations” implies the need for a threshold value that qualifies as “significant”. For example, what is a high enough density of a VME indicator or diversity of species for a site to be considered a VME?

**Figure 10:**
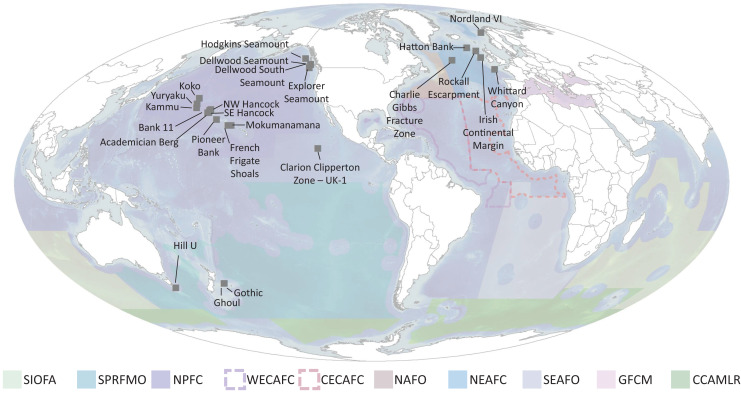
Locations of imagery data used for Question 4 (labelled points) and RFMO/As (colored regions) evaluated in this study. SIOFA, Southern Indian Ocean fisheries agreement region; SPRFMO, South Pacific regional fisheries management organisation; NPFC, North Pacific fisheries commission; WECAFC, Western Central Atlantic fishery commission; CECAF, fishery committee for the Eastern Central Atlantic; NAFO, North Atlantic fisheries organisation; NEAFC, North East Atlantic fisheries commission; SEAFO, South East Atlantic fisheries organisation; GFCM, general fisheries commission for the mediterranean; CCAMLR, commission for the conservation of antarctic marine living resources.

Ideally, defined thresholds would be based on *in situ* measurements to determine the functional significance of different densities of each taxon and the spatial extent of each species, since densities are taxon dependent and vary among regions due to abiotic conditions (*e.g*., depth, productivity regime) (OSPAR Commission, 2010a, 2010c, 2009). Currently however, only a few studies have started to quantify this in specific geographic regions and only for specific species (see section on “Towards Density Thresholds Related to Ecosystem Function”). Until more data are available, the next best approach is to address three questions: (1) What is the natural range of densities that VME taxa occur in? (2) What is the natural range of taxa richness that is observed in a single image? And (3) What portion of these ranges should be considered a VME? To address these questions, authors with available images each took 50 images at random from each of their study sites. These were selected from among images that included benthic megafauna. Using the list of VME taxa in Table 2, the number of each taxon in each image was counted. Then for each image, the expert gave their opinion on whether this image was a VME, in the format of ‘Yes’, ‘No’, or ‘Maybe’. Additional metadata collected include site name, depth, image area, % hard substrate, camera type, and camera resolution. Results were compiled from ten laboratories for 27 sites encompassing the North Atlantic, the North Pacific and the South Pacific (Table 3, Fig. 10 and Table S1), giving a total of 1,273 images. Work in the Papahānaumokuākea Marine National Monument was permitted under permit #PMNM-2014-028 and #PMNM-2016-021.

**Table 3:** Summary of available density data studies used in this analysis.

Region	Number of studies	Number of study sites	Areas
North Pacific	3	15	Northwestern Hawaiian Islands, Emperor Seamount Chain, Northeast Pacific Seamounts, Clarion-Clipperton Zone
Northeast Atlantic	4	6	Norwegian shelf, Irish canyons, Whittard Canyon, Rockall Bank, Hatton Bank
North Atlantic	1	3	Charlie Gibbs Fracture Zone
Southwest Pacific	2	3	Small seamounts east of New Zealand, Seamounts south of Tasmania
**Total**	**10**	**27**	

Data were analyzed as density and taxon richness per unit area using the image area to convert counts to density values. The range of image areas was large, 0.25–50 m^2^ but with 38 images having an area <1 m^2^. Analyses were therefore caried out on the 1,235 images of >1 m^2^ area to avoid inappropriate density extrapolations. R code used for the analyses can be found at https://github.com/bexeross/Baco-et-al-Anova-plots.git.

As abundance generally decreases with increasing depth (Rex & Etter, 2010), it might be expected that density would also decrease with depth and could confound the data analyses. A regression of observed density with depth (image range 100–4,176 m) showed no relationship (*p* = 0.6678), so depth was not included in further analyses. Similarly, densities might vary on hard substrates *vs* soft substrates. A regression of percent soft substrate *vs* overall density showed there was also no difference in the observed values by substrate type (*p* = 0.3432).

**Figure 11:**
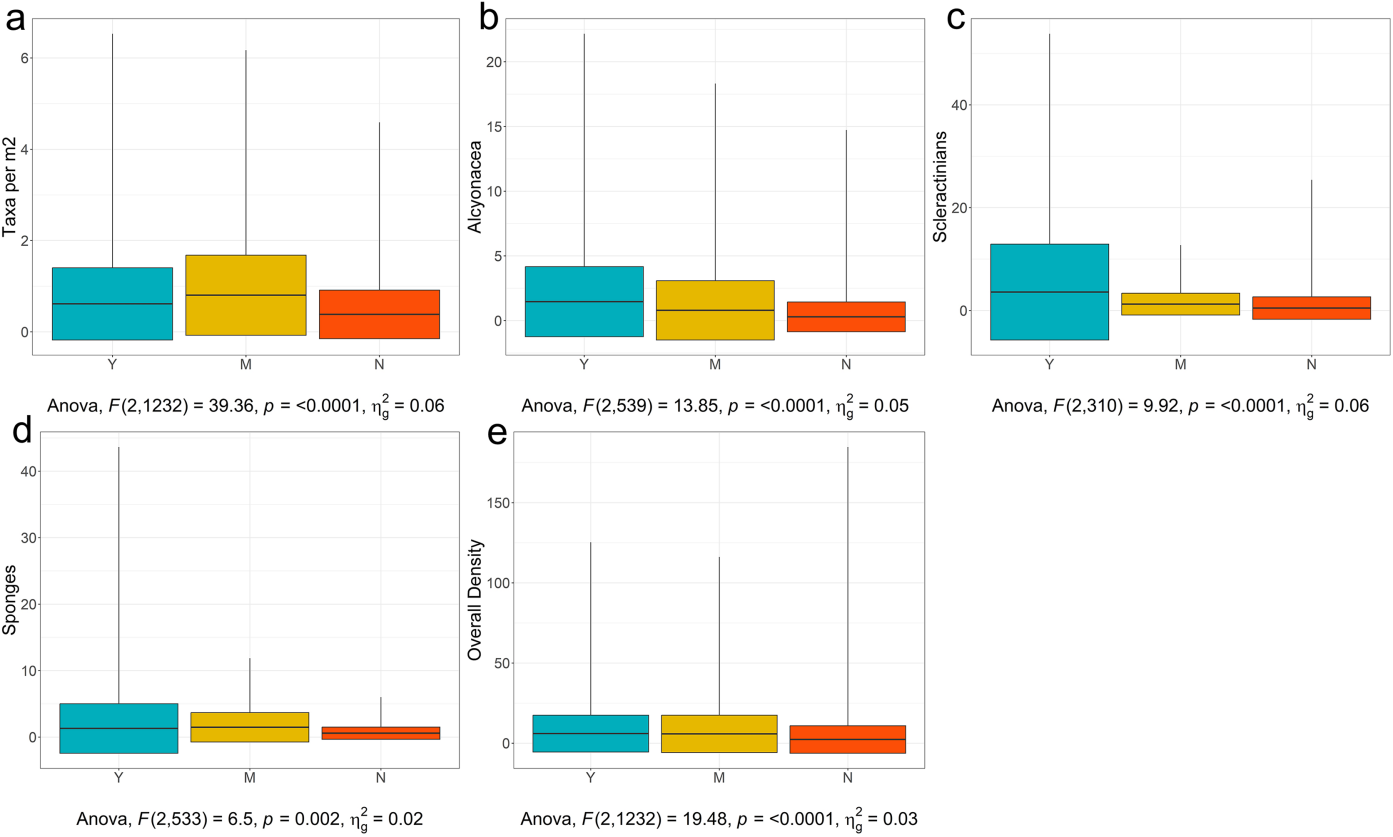
Boxplots of YMN (Yes, Maybe, No) from images examined for VMEs. (A)Number of Taxa per Image, (B) Density of Alcyonacea, (C) Density of Scleractinia, (D) Density of Porifera, and (E) Overall Density. Global one-way ANOVA results are provided below each figure. Figures for additional taxa are available in Fig. S1.

With these potential confounding factors accounted for, the first threshold in Fig. 9 is for number of taxa. The observed range of VME taxon richness per image was 1–9 taxa, giving a range of 0.02–6.53 taxa per m^2^ with a mean of 0.54 (see Fig. 11A for taxa per m^2^ visualization and Table S2 for summary statistics for all tested categories). The range of observed taxon richness values per m^2^ were overlapping for all of three VME designation categories. Regardless, a simple one-way analysis of variance (see Table S3 for ANOVA results for all analyses) showed that there is a statistically significant difference between VME designation choices (*p* < 0.001, Fig. 11A, although *p* < 0.01 for ***Yes*** *vs Maybe*, and ***Yes*** *vs No* see Table S4 for Tukey HSD results for all analyses). *No* had a mean of 0.45 ± 0.7 taxa per m^2^, *Maybe* a mean of 0.98 ± 1.4 taxa per m^2^, and ***Yes*** a mean of 0.69 ± 1.1 taxa per m^2^. The disparity between ***Yes*** and *Maybe* values possibly reflect other factors such as which taxa are co-occurring, the size and density of the taxa in question, and whether all taxa are agreed upon across all RFMO/As.

Working at a coarse taxonomic level, for most taxa, there was a statistically significant difference in densities for ***Yes*** *vs No*. For example, alcyonaceans had a significant difference for all three choices (*p* < 0.0001, Fig. 11B) with the mean for ***Yes*** being 1.47 ± 2.7 individuals per m^2^ and *No* being 0.29 ± 1.2 individuals per m^2^.

For analytical simplicity, Scleractinian corals that form a reef structure were annotated as counts where feasible by detecting distinct coral heads or isolated colonies. However, percentage cover (not considered here) is also an important metric when quantifying cold-water coral reef VMEs. Scleractinians were further complicated by the fact that some regions consider cup corals to be VME indicators while others do not. As a result, values in the *No* category ranged up to 25.4 individuals per m^2^. Despite the broad range of values, Scleractinians showed significant differences between ***Yes***, with a mean of 3.59 ± 9.3 individuals per m^2^, and 0.48 ± 2.18 individuals per m^2^ for *No* (*p* < 0.0001, Fig. 11C, Tables S3 and S4).

Pairwise tests for sponges (Porifera) showed a significant difference between ***Yes*** and *No* (*p* = 0.0175, Fig. 11D, Tables S3 and S4), with ***Yes*** having a mean of 1.29 ± 3.7 individuals per m^2^, and *No* having a mean of 0.58 ± 0.9 individuals per m^2^. However, there was no significant difference between ***Yes*** and *Maybe* (mean of 1.48 ± 2.21). Overlap may have arisen because experts from different regions have different opinions on encrusting *vs* upright sponges, or on the critical density of sponges that constitute a VME. Sponges are also very diverse in shape and size with larger individuals potentially more likely to be associated with VMEs (*e.g*., NAFO lists large-sized sponges specifically, NAFO, 2021), however, larger taxa will also occur in lower densities which may further confound this assessment. Figures and analyses for additional taxa are provided in Fig. S1 and Tables S2–S4.

The final threshold in Fig. 9 is for the density of all VME taxa combined. The general test was significant (*p* < 0.0001, Fig. 11E and Table S3). The mean for ***Yes*** was 6.00 ± 11.5 individuals per m^2^. The mean for *No* was 2.32 ± 8.6 individuals per m^2^. However again there was no difference between ***Yes*** and *Maybe* (mean 5.81 ± 11.7, Table S4). This result may reflect the issues pointed out above where small individuals can be highly numerous but may not be considered a clear VME indicator to some experts and regions.

This is a coarse approach, but is a start for developing threshold density and diversity metrics for designating VMEs, and will require significant refinement before realistic or consistent thresholds can be established. However, from this exercise we can infer several points. The first is that there is generally a difference between the means for deciding ***Yes*** a site is a VME and *No* a site is not a VME, indicating that there is the potential to develop consistent thresholds across regions based on expert consensus. The density values for a ***Yes*** *vs No* for individual taxa, however, vary widely among groups, making it unrealistic to set a threshold density that will work across all taxa. Thus, taxon thresholds will need to be calculated on a taxon-specific and potentially a regional basis, with any calculations ideally taking taxon size into account. Our study sites were clustered in the North Atlantic and North Pacific, additional information from the South Atlantic, South Pacific, Southern Ocean and Indian Ocean is of paramount importance in order to form robust, evidence-based thresholds on both a global and regional scale (Fig. 10).

## Open questions and next steps

### Areal extent

This effort is a work in progress and represents the first step in the process of determining an objective, quantitative method for identifying VMEs from images. However, many more questions need to be addressed to advance this process *e.g*., advancing beyond single images. While an image of ~10 m^2^ (range of study images 0.25–50 m^2^) may show high densities of taxa, this tiny area relative to the total management area makes it challenging to use a single image to justify management actions such as area closures. Furthermore, some VMEs cannot be discerned from a single image alone due to the dominance of larger taxa or having lower natural densities (*e.g*., Figs. 8A and 8B). A greater number of images would have a better chance of capturing natural variability in distribution and density to both initiate management actions and ensure that areas dominated by large taxa or low-density VMEs are also identified.

On the positive side, single images and video frames are rarely taken in isolation, generally even drop cameras are deployed multiple times at a survey location. If multiple single images from a location are determined to depict VMEs, then there would be great support for management action. However, the issue remains that there is a need to determine how many images over what area need to be taken and what proportion of those need to depict VMEs. Thus, the next step proposed for this process is to develop standards for multiple image/video assessments to capture the areal extent of VMEs.

This is not straightforward, since setting thresholds for VME areal extent could be based on: VME community patch sizes (*e.g*., the average size of a reef), average densities of lower density VME indicators (*e.g*., *Umbellula encrinus* Linnaeus, 1758 giant sea pen communities in Norway may only reach densities of 6.4 individuals every 100 m^2^; Gonzalez-Mirelis & Buhl-Mortensen, 2015), minimum viable community patch sizes (*e.g*., >25 m^2^ is suggested by OSPAR as the minimum areal extent for a biotope, OSPAR, 2008a, 2008b), management practicalities (*e.g*., utilizing current RFMO/A move-on rule distances, trawl haul areas, or minimum viable MPA sizes as buffer areas of search), or identifying geomorphological features that host VMEs (*e.g*., seamounts, which could be considered as VMEs themselves, *sensu* Watling & Auster, 2017).

Many of these approaches have limitations. A VME community patch size-based approach would need to recognize that the natural range of patch sizes appears to be both dominant-taxon- and location-dependent. For example, cold-water coral reefs dominated by *Solenosmilia variabilis* have been predicted to be between 625 m^2^–0.425 km^2^ in size in New Zealand (Rowden et al., 2017) but 0.02–1.16 km^2^ in Tasmania (Williams et al., 2020a). Boreal Ostur sponge aggregations in Norway have been measured at >50 km^2^ with empty gaps of <30 m (Kutti, Bannister & Fosså, 2013), while glass sponge reefs in Canada may be only 35–72 m in diameter (Chu & Leys, 2010). Should a review of patch sizes be undertaken, potentially a minimum viable distance could be determined to identify the minimum number of images at X meters apart needed to be sure that management action could be worth initiating. However, the low-density VMEs that could not be identified from a single image may continue to be overlooked by failing to survey a wide enough area. Meanwhile, move-on rule distances or minimum viable MPA sizes are RFMO/A dependent, and geomorphological features may capture some VMEs, but soft-bottom VME communities cannot be delimited in this fashion (*e.g*., xenophyophore fields and sea pen aggregations). It is therefore likely that any VME areal extent standard that could be developed would need to be flexible, describing multiple search techniques and listing multiple criteria.

### Confidence index

Another useful step would be to develop a Confidence Index *e.g*., tied to the number of images in an area that represent a VME. Confidence is commonly used to assess the accuracy or uncertainty of a method or product and can be assessed based on a range of factors, including data quality and data deficiency (Wallace et al., 2011). Morato et al. (2018) developed a multi-criteria assessment to evaluate the likelihood of VME presence in the North Atlantic. This approach was based on available VME indicator and habitat data from the ICES VME database (ICES, 2016), with outputs mapped on a 0.05° × 0.05° grid cell scale. As part of this method, a measure of confidence was included based on four criteria: the survey method (with visual surveys scoring higher than trawl surveys or other survey methods such as acoustic data); the number of surveys in the area (grid cell); the survey time period; and the age of the last survey in the area. Final scores were assigned to grid cells as either ‘High’, ‘Medium’ or ‘Low’ confidence, and could be mapped alongside the likelihood of VME presence to present a visual representation of outputs. Similar methods, along with an evaluation of the existing confidence index approaches, could be considered within a global standard for any VME assessment method.

### Towards density thresholds related to ecosystem function

A key next step in developing VME indicator density thresholds is continuing the study of ecosystem function relative to VME indicator density. Links between structure forming VME indicator taxa and enhanced biodiversity (Beazley et al., 2013; Jonsson et al., 2004; Henry & Roberts, 2007; Price et al., 2019), fisheries species (Foley et al., 2010; Stone, 2014; Rooper, Goddard & Wilborn, 2019; Price et al., 2019; Henderson, Huff & Yoklavich, 2020) and ecosystem functioning (Kutti, Bannister & Fosså, 2013; de Clippele et al., 2021) have been well documented. However, few studies have quantified VME indicator density with associated diversity and ecosystem functioning. Understanding when VME indicator become dense enough to form an influential habitat (and presumably a VME), can help underpin marine spatial management solutions through objective definitions of habitats (Bullimore, Foster & Howell, 2013), and predicting the spatial extent of VME indicator taxa/habitat (Rowden et al., 2017; Williams et al., 2020a).

Most studies defining VME indicator density thresholds focus on natural density ranges and omit quantitative analyses of associated biodiversity or ecosystem function. For example, Vertino et al. (2010) used percent coverage to distinguish mound (40–66%) and inter-mound (6–9%) reef habitats with 20–40% coverage of live or dead coral delimiting “coral framework” in the Mediterranean Sea (Vertino et al., 2010). Rogers et al. (2013) suggested coral colony densities should reach >10 times background densities, and usually >0.1 colonies per m^2^. Using this approach, Bullimore, Foster & Howell (2013) assessed the background density in their area of study and found a value of >0.47 colonies m^2^ would be required to achieve >10 times the background density. However, this value is relative and therefore area specific.

Some studies have started to link ecological function and density thresholds though. Henry & Roberts (2014a, 2014b) in reviewing OSPAR Threatened and/or declining habitat definitions for coral gardens and deep sea sponge aggregations, assessed published data against a series of criteria including density and ecological function. Coral densities required that VME indicators were at least “frequent” on the SACFOR scale (Strong & Johnson, 2020) in an image, video, or sample, while ecological function was considered high when other species co-occurred in high frequencies, or non-coral taxa characterized at least 50% of the assemblages. Sponge aggregations, used OSPAR density thresholds, the SACFOR scale, or the NEAFC move-on threshold of 400 kg (NEAFC, 2014), while ecological function required presence of listed associated fauna (as outlined in OSPAR Commission, 2010b) or that a SIMPER analysis (Clarke & Warwick, 2001) highlighted other taxa as characteristic of the assemblage.

Additional literature has focused on connecting the density of VME indicators and the diversity of associated fauna. For example, Beazley et al. (2015) used imagery to identify that the largest turnover in megafaunal community composition in NW Atlantic sponge grounds occurred when the sponges reached 15 individuals m^2^. Price et al. (2019) used 3D photogrammetry on a scale of tens of meters to link structural complexity and biodiversity, finding areas of high structural complexity and coral coverage above 30% harbored distinct and more diverse communities. While Rowden et al. (2020) posited thresholds for “significant concentrations” supporting “high diversity” in *Solenosmilia variabilis* reefs near New Zealand as 24.5–28% cover of framework-building coral or a density of “live coral heads” of 0.11–0.14 (over areas of 50, 25 m^2^ in video) or 0.85 coral heads per m^2^ (in 2 m^2^ still images).

Imagery data is starting to play a critical role in assessing ecosystem function, but a greater number of studies need to be completed before threshold guidelines can be developed that directly tie species composition, abundance, or density to ecosystem function for most VME indicators. In the meantime, imagery data provides a more accurate picture of species composition, density, and functional importance than trawling surveys do, so images will be a more accurate way to determine VME ecosystem function-density thresholds.

### Habitat suitability modeling

Another tool used for the designation of VMEs is habitat suitability modeling. Obtaining images or bycatch samples of VME indicator taxa may confirm their presence, but the proportion of the seafloor that has been observed or sampled to date is <0.001% (Stel, 2021). Habitat Suitability Modelling (HSM, aka Ecological Niche Modeling) is one way to fill in the gaps and provide an objective prediction of where VMEs may exist in the unexplored regions of the world’s oceans. HSM refers to the use of computer algorithms to model the mathematical relationship between occurrences of a species/habitat and its preferred environmental conditions such that its spatial distribution can be predicted in unsampled areas with environmental data (Vierod, Guinotte & Davies, 2014).

The use of HSM as evidence for the management of VMEs was endorsed in 2016 by UNGA resolution 71/123 (§180-181) and guidelines for their use remain under development by management bodies (ICES, 2021). To date, HSM has been used *inter alia* to provide a basis for spatial management planning (Rowden et al., 2019), estimate MPA effectiveness against percentage targets (Ross & Howell, 2013), cross-reference VME distribution and fishery activity (Jackson et al., 2014), locate potential higher density VME thresholds and hotspots (Rowden et al., 2017, Gonzalez-Mirelis et al., 2021), predict pre-fishing baseline densities of VME indicator taxa (Downie et al., 2021), predict potential changes in VME indicator distribution under climate projections (Morato et al., 2020), and to combine with dispersal estimates to identify isolated VME populations (Ross, Wort & Howell, 2019).

Most commonly, HSM is applied to species (aka Species Distribution Models, SDMs, *e.g*., for a VME indicator taxon) but these predictions do not necessarily capture the distribution of the community that taxon is associated with, nor the specific areas where a species may form more complex habitat, *e.g*., deep-sea scleractinian coral reefs (Howell et al., 2011). However, there are growing efforts to apply HSM to communities/biotopes (Ferrier & Guisan, 2006; Howell et al., 2016), to density of structure-forming taxa, (*e.g*., Rooper et al., 2016, 2018), or traits (*e.g*., Murillo et al., 2020), multiple taxa simultaneously (*e.g*., Joint Species Distribution Modelling, Warton et al., 2015), or to intersect multiple stacked SDMs (*e.g*., Lyons et al., 2020): approaches which may be better suited to capturing VME extent and distribution.

It is important to note that the modelling approaches described above are fallible, with issues stemming from sampling bias, positioning errors, combining datasets with different qualities, coverage and resolution of environmental data, taxonomic resolution, unaccounted for drivers/limiters of distribution, *etc*. all contributing to potential errors in model predictions (Vierod, Guinotte & Davies, 2014). Indeed, field validation studies have indicated deep-sea HSMs based on low resolution global bathymetry performed poorly (Anderson et al., 2016), highlighting the need for suitable high-resolution underpinning predictor datasets. Key elements of models to be useful for VME identification are to quantify uncertainty in available predictor variables, ensure variables are ecologically significant, use abundance or biomass data rather than presence/absence, and increase taxonomic resolution to ecologically relevant levels (Bowden et al., 2021). It is therefore necessary to request and utilize any information on model uncertainty to temper model interpretations. Ground-truthing of models using imaging surveys will also improve their accuracy (Rooper et al., 2016, 2018; Winship et al., 2020), or lead to their rejection for VME designation use (*e.g*., Anderson et al., 2016). A close interaction between modelers and stakeholders can help to ensure that HSM and other forms of modelling are properly applied and interpreted to benefit marine management needs (Villero et al., 2016).

## Conclusions and management recommendations

The goals of this international effort were to build a community consensus for a quantitative approach for determining what constitutes a VME from imagery data. This article represents the first step in that process, providing a framework for VME identification from single images or single video frames. Further work will focus on the use of multiple images and metrics of confidence in VME designation from imagery. This work has highlighted several management recommendations:

First is a need for consistency among RFMO/As on a minimum list of taxa that should be considered VME indicators. The current variability in designations among regions impedes development of an international consensus on VME designations and therefore risks damaging these ecosystems. This global list would make the process of designation of VMEs more efficient and its continual development would be imperative given ongoing exploration. Beyond fisheries, having such a consensus list could benefit management in other sectors, including informing biodiversity conservation efforts in ABNJ, deep-seabed mining, and oil and gas exploration.

Secondly, this study demonstrates that VMEs can clearly and confidently be identified from imagery. While more extensive surveys with multiple images are always preferable, certain VMEs can even be identified at the scale of a single image or frame. Imagery data is increasingly becoming available, and does not carry the same limitations of the current common approach of using fisheries bycatch thresholds to identify VMEs (see Introduction). Therefore, the authors recommend that RFMO/As should consider adopting guidance that will allow image surveys of VMEs as a viable alternative for detecting VMEs in addition to, or even in place of fisheries bycatch data, particularly for impact assessments of previously unfished areas. Images that cover a larger area of seafloor, while still retaining adequate resolution of fauna for identifications, will be the most valuable for this approach.

Relatedly, the authors’ third recommendation is for the inclusion of imagery as a requirement for fisheries impact assessments (IAs) of benthic areas. The less destructive and more accurate nature of imagery data recommends it above trawl-based IAs. The FAO Guidelines state that: “an impact assessment should address, *inter alia*…identification, description and mapping of VMEs known or likely to occur in the fishing area” (FAO, 2009, paragraph 47). A standardized IA imagery survey approach could be developed to provide adequate imagery to determine densities, confidence, and areal extent indexes. There are already recommendations for standardized survey methods and annotation tools, *e.g*., GOSSIP (Woodall et al., 2018), SACFOR (Strong & Johnson, 2020), and Australia’s monitoring field manuals (Przeslawski et al., 2018). These efforts could provide a basis for the development of tools standardized across regions.

Finally, the term “VME” and the requirements for their protection from SAIs were developed specifically in the context of high-seas fisheries (FAO, 2009). However, fishing is not the only activity that can result in SAIs to benthic marine communities (Ragnarsson et al., 2016). Other human activities that may lead to damage of VMEs include deep-sea mining (Martins et al., 2018; Ragnarsson et al., 2016; Gollner et al., 2017; Ramirez-Llodra et al., 2020; Levin, Amon & Lily, 2020; Amon et al., 2022), tailing placement (Vare et al., 2018; Ramirez-Llodra et al., 2011), oil/gas extraction (Larsson & Purser, 2011; Cordes et al., 2016; Amon et al., 2017; Luter et al., 2019; Vad et al., 2018, 2020), accidental oil spills and dispersant used for clean ups (Bytingsvik et al., 2020; Etnoyer et al., 2016; Girard & Fisher, 2018; Luter et al., 2019; Vad et al., 2020), mairine debris and plastic pollution (Pham et al., 2014a, 2014b; Taylor et al., 2016; Chapron et al., 2018; Mouchi et al., 2019; Amon et al., 2020; de Oliveira Soares et al., 2020), and climate change (*e.g*., Sweetman et al., 2017). Thus, the fourth recommendation is for visual surveys to be incorporated into IAs for all industries working in the deep sea and for the VME designation and criteria to be used by all industries to develop management tools to holistically avoid SAIs to benthic communities.

## Supporting information captions

### Detailed analyses results from threshold investigations

All analyses were performed in R with the results forming the basis of threshold conclusions relating to Question 4 (*What are the thresholds (density or diversity) that need to be met to characterize a single image as a VME?)* R code used for the analyses can be found at https://github.com/bexeross/Baco-et-al-Anova-plots.git.

Supplemental Figure 1. Boxplots of YMN (Yes, Maybe, No) for Taxa per m^2^ and additional taxa.

Supplemental Table 1. Raw data used for threshold investigations in Question 4.

Supplemental Table 2. Summary statistics for YMN (Yes, Maybe, No) decisions per category discussed in the text in Question 4 across all images with >1 m^2^ area.

Supplemental Table 3. Anova (stats::aov) results tables for YMN (Yes, Maybe, No) decisions per category discussed in the Question 4 across all images with >1 m^2^ area.

Supplemental Table 4. Tukey HSD (rstatix::tukey_hsd) results tables for YMN (Yes, Maybe, No) decisions per category discussed in Question 4 across all images with >1 m^2^ area.

## Supplemental Information

10.7717/peerj.16024/supp-1 Supplemental Information 1 Raw data used for threshold investigations in Question 4.

10.7717/peerj.16024/supp-2 Supplemental Information 2 Supplemental Figure 1 and Tables 2–4.

## Additional Information and Declarations

### Data Availability

The following information was supplied regarding data availability: The raw data related to the analyses in Question 4 are included as Table S1.
