# Supplementary material for: Towards a scientific community consensus on designating Vulnerable Marine Ecosystems from imagery
Source: PeerJ. 2023 Oct 12;11:e16024. doi: 10.7717/peerj.16024 (PMC10576969; doi:10.7717/peerj.16024)
Supplement: Supplemental Information 2 [file peerj-11-16024-s002.docx]

# Supplemental Materials

# Detailed analyses results from threshold investigations

All analyses were performed in R with the following results forming the basis of threshold conclusions relating to Question 4 (***For the last three elements of the flow chart, what are the thresholds that need to be met for VME designation?****)*.

## Supplemental Figure 1. Boxplots of YMN (Yes, Maybe, No) for Taxa per m^2^ and additional taxa.


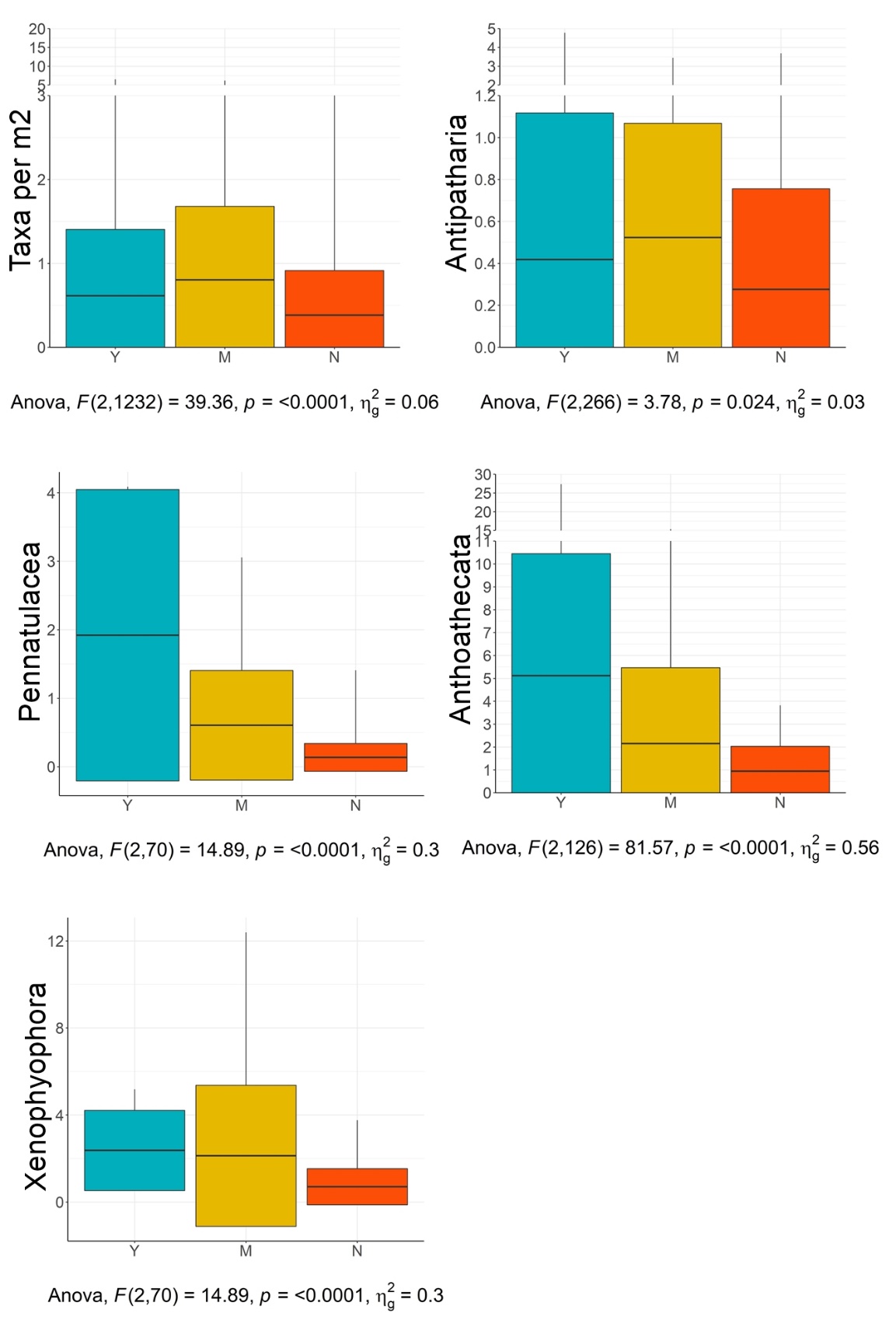


## Supplemental Table 2. Summary statistics for YMN (Yes, Maybe, No) decisions per category discussed in the text in Question 4 across all images with >1m^2^ area.

|  | VME_YMN | mean | sd | n | min | max |
| --- | --- | --- | --- | --- | --- | --- |
|  | <fctr> | <dbl> | <dbl> | <int> | <dbl> | <dbl> |
| Overall Density | Y | 6.00 | 11.47 | 293 | 0.17 | 125.32 |
|  | M | 5.81 | 11.65 | 290 | 0.10 | 116.13 |
|  | N | 2.32 | 8.57 | 652 | 0.03 | 184.72 |
| Total Taxa | Y | 3.93 | 1.62 | 293 | 1.00 | 9.00 |
|  | M | 3.61 | 1.69 | 290 | 1.00 | 9.00 |
|  | N | 2.16 | 1.24 | 652 | 1.00 | 8.00 |
| Taxa per m2 | Y | 0.61 | 0.79 | 293 | 0.05 | 6.53 |
|  | M | 0.80 | 0.88 | 290 | 0.05 | 6.17 |
|  | N | 0.38 | 0.53 | 652 | 0.02 | 4.59 |
| Scleractinia | Y | 3.59 | 9.32 | 73 | 0.07 | 53.83 |
|  | M | 1.25 | 2.12 | 99 | 0.07 | 12.72 |
|  | N | 0.48 | 2.18 | 141 | 0.04 | 25.40 |
| Alcyonacea | Y | 1.47 | 2.71 | 224 | 0.02 | 22.17 |
|  | M | 0.80 | 2.29 | 150 | 0.05 | 18.31 |
|  | N | 0.29 | 1.15 | 168 | 0.03 | 14.73 |
| Pennatulacea | Y | 1.92 | 2.13 | 4 | 0.09 | 4.09 |
|  | M | 0.60 | 0.80 | 26 | 0.05 | 3.06 |
|  | N | 0.14 | 0.20 | 43 | 0.03 | 1.41 |
| Antipatharia | Y | 0.42 | 0.70 | 97 | 0.02 | 4.78 |
|  | M | 0.52 | 0.55 | 91 | 0.03 | 3.44 |
|  | N | 0.28 | 0.48 | 81 | 0.03 | 3.68 |
| Porifera | Y | 1.29 | 3.72 | 177 | 0.05 | 43.64 |
|  | M | 1.48 | 2.21 | 158 | 0.03 | 11.87 |
|  | N | 0.58 | 0.92 | 201 | 0.02 | 6.02 |
| Xenophyopora | Y | 2.37 | 1.84 | 7 | 0.52 | 5.18 |
|  | M | 2.13 | 3.24 | 38 | 0.07 | 12.40 |
|  | N | 0.70 | 0.83 | 34 | 0.06 | 3.76 |
| Anthoathecata | Y | 5.11 | 5.34 | 48 | 0.20 | 27.33 |
|  | M | 2.15 | 3.31 | 29 | 0.07 | 15.34 |
|  | N | 0.95 | 1.08 | 24 | 0.07 | 3.82 |

Supplemental Table 3. Anova (stats::aov) results tables for YMN (Yes, Maybe, No) decisions per category discussed in the Question 4 across all images with >1m^2^ area.

Significance codes: 0 ‘***’ 0.001 ‘**’ 0.01 ‘*’ 0.05 ‘.’ 0.1 ‘ ’ 1

|  |  | Df | SumSq | MeanSq | Fvalue | Pr(>F) |  |
| --- | --- | --- | --- | --- | --- | --- | --- |
| Overall Density | data.cln$VME_YMN | 2 | 3968 | 1984 | 19.48 | 4.71E-09 | *** |
|  | Residuals | 1232 | 125509 | 101.9 |  |  |  |
|  | --- |  |  |  |  |  |  |
|  |  |  |  |  |  |  |  |
|  |  |  |  |  |  |  |  |
|  |  | Df | SumSq | MeanSq | Fvalue | Pr(>F) |  |
| Total Taxa | data.cln$VME_YMN | 2 | 813.2 | 406.6 | 193.3 | <2e-16 | *** |
|  | Residuals | 1232 | 2591.9 | 2.1 |  |  |  |
|  | --- |  |  |  |  |  |  |
|  |  |  |  |  |  |  |  |
|  |  |  |  |  |  |  |  |
|  |  | Df | SumSq | MeanSq | Fvalue | Pr(>F) |  |
| Taxa per m2 | data.cln$VME_YMN | 2 | 37.7 | 18.827 | 39.36 | <2e-16 | *** |
|  | Residuals | 1232 | 589.3 | 0.478 |  |  |  |
|  | --- |  |  |  |  |  |  |
|  |  |  |  |  |  |  |  |
|  |  |  |  |  |  |  |  |
|  |  | Df | SumSq | MeanSq | Fvalue | Pr(>F) |  |
| Scleractinia | data.sc$VME_YMN | 2 | 471 | 235.52 | 9.923 | 6.65E-05 | *** |
|  | Residuals | 310 | 7357 | 23.73 |  |  |  |
|  | --- |  |  |  |  |  |  |
|  |  |  |  |  |  |  |  |
|  |  |  |  |  |  |  |  |
|  |  | Df | SumSq | MeanSq | Fvalue | Pr(>F) |  |
| Alcyonacea | data.oc$VME_YMN | 2 | 135.5 | 67.77 | 13.85 | 1.36E-06 | *** |
|  | Residuals | 539 | 2637.4 | 4.89 |  |  |  |
|  | --- |  |  |  |  |  |  |
|  |  |  |  |  |  |  |  |
|  |  |  |  |  |  |  |  |
|  |  | Df | SumSq | MeanSq | Fvalue | Pr(>F) |  |
| Pennatulacea | data.pn$VME_YMN | 2 | 13.33 | 6.666 | 14.89 | 4.10E-06 | *** |
|  | Residuals | 70 | 31.34 | 0.448 |  |  |  |
|  | --- |  |  |  |  |  |  |
|  |  |  |  |  |  |  |  |
|  |  |  |  |  |  |  |  |
|  |  | Df | SumSq | MeanSq | Fvalue | Pr(>F) |  |
| Antipatharia | data.an$VME_YMN | 2 | 2.61 | 1.3073 | 3.777 | 0.0241 | * |
|  | Residuals | 266 | 92.08 | 0.3462 |  |  |  |
|  | --- |  |  |  |  |  |  |
|  |  |  |  |  |  |  |  |
|  |  |  |  |  |  |  |  |
|  |  | Df | SumSq | MeanSq | Fvalue | Pr(>F) |  |
| Porifera | data.sp$VME_YMN | 2 | 82 | 41.1 | 6.496 | 0.00163 | ** |
|  | Residuals | 533 | 3372 | 6.33 |  |  |  |
|  | --- |  |  |  |  |  |  |
|  |  |  |  |  |  |  |  |
|  |  |  |  |  |  |  |  |
|  |  | Df | SumSq | MeanSq | Fvalue | Pr(>F) |  |
| Xenophyopora | data.xe$VME_YMN | 2 | 41.7 | 20.825 | 3.663 | 0.0303 | * |
|  | Residuals | 76 | 432.1 | 5.685 |  |  |  |
|  | --- |  |  |  |  |  |  |
|  |  |  |  |  |  |  |  |
|  |  |  |  |  |  |  |  |
|  |  | Df | SumSq | MeanSq | Fvalue | Pr(>F) |  |
| Anthoathecata | data.st$VME_YMN | 2 | 329 | 164.52 | 9.64 | 0.000151 | *** |
|  | Residuals | 98 | 1672 | 17.07 |  |  |  |
|  | --- |  |  |  |  |  |  |

## Supplemental Table 4. Tukey HSD (rstatix::tukey_hsd) results tables for YMN (Yes, Maybe, No) decisions per category discussed in Question 4 across all images with >1m^2^ area.

Significance codes: 0 ‘***’ 0.001 ‘**’ 0.01 ‘*’ 0.05 ‘.’ 0.1 ‘ ’ 1

|  |  | | term | group1 | group2 | null.value | estimate | conf.low | conf.high | p.adj | p.adj.signif |
| --- | --- | --- | --- | --- | --- | --- | --- | --- | --- | --- | --- |
| Overall Density | |  | <chr> | <chr> | <chr> | <dbl> | <dbl> | <dbl> | <dbl> | <dbl> | <chr> |
|  | 1 | | data.cln$VME_YMN | Y | M | 0 | -0.19107 | -2.1529 | 1.770758 | 9.72E-01 | ns |
|  | 2 | | data.cln$VME_YMN | Y | N | 0 | -3.68319 | -5.34897 | -2.01741 | 7.42E-07 | **** |
|  | 3 | | data.cln$VME_YMN | M | N | 0 | -3.49212 | -5.16384 | -1.82041 | 3.22E-06 | **** |
|  |  | |  |  |  |  |  |  |  |  |  |
|  |  | | term | group1 | group2 | null.value | estimate | conf.low | conf.high | p.adj | p.adj.signif |
| Total Taxa |  | | <chr> | <chr> | <chr> | <dbl> | <dbl> | <dbl> | <dbl> | <dbl> | <chr> |
|  | 1 | | data.cln$VME_YMN | Y | M | 0 | -0.31798 | -0.59991 | -0.03606 | 0.0224 | * |
|  | 2 | | data.cln$VME_YMN | Y | N | 0 | -1.76882 | -2.0082 | -1.52944 | 0 | **** |
|  | 3 | | data.cln$VME_YMN | M | N | 0 | -1.45084 | -1.69107 | -1.2106 | 0 | **** |
|  |  | |  |  |  |  |  |  |  |  |  |
|  |  | | term | group1 | group2 | null.value | estimate | conf.low | conf.high | p.adj | p.adj.signif |
| Taxa per m2 |  | | <chr> | <chr> | <chr> | <dbl> | <dbl> | <dbl> | <dbl> | <dbl> | <chr> |
|  | 1 | | data.cln$VME_YMN | Y | M | 0 | 0.18972 | 0.055295 | 0.324146 | 2.74E-03 | ** |
|  | 2 | | data.cln$VME_YMN | Y | N | 0 | -0.23012 | -0.34426 | -0.11598 | 7.44E-06 | **** |
|  | 3 | | data.cln$VME_YMN | M | N | 0 | -0.41984 | -0.53438 | -0.30529 | 0.00E+00 | **** |
|  |  | |  |  |  |  |  |  |  |  |  |
|  |  | | term | group1 | group2 | null.value | estimate | conf.low | conf.high | p.adj | p.adj.signif |
| Scleractinia |  | | <chr> | <chr> | <chr> | <dbl> | <dbl> | <dbl> | <dbl> | <dbl> | <chr> |
|  | 1 | | data.sc$VME_YMN | Y | M | 0 | -2.34693 | -4.11688 | -0.57698 | 5.55E-03 | ** |
|  | 2 | | data.sc$VME_YMN | Y | N | 0 | -3.10933 | -4.76362 | -1.45504 | 3.95E-05 | **** |
|  | 3 | | data.sc$VME_YMN | M | N | 0 | -0.7624 | -2.26677 | 0.741966 | 4.58E-01 | ns |
|  |  | |  |  |  |  |  |  |  |  |  |
|  |  | | term | group1 | group2 | null.value | estimate | conf.low | conf.high | p.adj | p.adj.signif |
| Alcyonacea |  | | <chr> | <chr> | <chr> | <dbl> | <dbl> | <dbl> | <dbl> | <dbl> | <chr> |
|  | 1 | | data.oc$VME_YMN | Y | M | 0 | -0.67199 | -1.22048 | -0.1235 | 1.15E-02 | * |
|  | 2 | | data.oc$VME_YMN | Y | N | 0 | -1.17468 | -1.70528 | -0.64408 | 8.35E-07 | **** |
|  | 3 | | data.oc$VME_YMN | M | N | 0 | -0.50269 | -1.08669 | 0.081317 | 1.08E-01 | ns |
|  |  | |  |  |  |  |  |  |  |  |  |
|  |  | | term | group1 | group2 | null.value | estimate | conf.low | conf.high | p.adj | p.adj.signif |
| Pennatulacea |  | | <chr> | <chr> | <chr> | <dbl> | <dbl> | <dbl> | <dbl> | <dbl> | <chr> |
|  | 1 | | data.pn$VME_YMN | Y | M | 0 | -1.31567 | -2.1762 | -0.45514 | 1.39E-03 | ** |
|  | 2 | | data.pn$VME_YMN | Y | N | 0 | -1.78433 | -2.62187 | -0.94678 | 8.20E-06 | **** |
|  | 3 | | data.pn$VME_YMN | M | N | 0 | -0.46866 | -0.8667 | -0.07062 | 1.70E-02 | * |
|  |  | |  |  |  |  |  |  |  |  |  |
|  |  | | term | group1 | group2 | null.value | estimate | conf.low | conf.high | p.adj | p.adj.signif |
| Antipatharia |  | | <chr> | <chr> | <chr> | <dbl> | <dbl> | <dbl> | <dbl> | <dbl> | <chr> |
|  | 1 | | data.an$VME_YMN | Y | M | 0 | 0.10457 | -0.0978 | 0.306944 | 0.444 | ns |
|  | 2 | | data.an$VME_YMN | Y | N | 0 | -0.14204 | -0.35076 | 0.066676 | 0.246 | ns |
|  | 3 | | data.an$VME_YMN | M | N | 0 | -0.24661 | -0.45844 | -0.03479 | 0.0178 | * |
|  |  | |  |  |  |  |  |  |  |  |  |
|  |  | | term | group1 | group2 | null.value | estimate | conf.low | conf.high | p.adj | p.adj.signif |
| Porifera |  | | <chr> | <chr> | <chr> | <dbl> | <dbl> | <dbl> | <dbl> | <dbl> | <chr> |
|  | 1 | | data.sp$VME_YMN | Y | M | 0 | 0.180476 | -0.46657 | 0.827519 | 0.789 | ns |
|  | 2 | | data.sp$VME_YMN | Y | N | 0 | -0.7103 | -1.31968 | -0.10092 | 0.0175 | * |
|  | 3 | | data.sp$VME_YMN | M | N | 0 | -0.89077 | -1.51933 | -0.26221 | 0.00266 | ** |
|  |  | |  |  |  |  |  |  |  |  |  |
|  |  | | term | group1 | group2 | null.value | estimate | conf.low | conf.high | p.adj | p.adj.signif |
| Xenophyopora |  | | <chr> | <chr> | <chr> | <dbl> | <dbl> | <dbl> | <dbl> | <dbl> | <chr> |
|  | 1 | | data.xe$VME_YMN | Y | M | 0 | -0.24458 | -2.58898 | 2.099825 | 0.966 | ns |
|  | 2 | | data.xe$VME_YMN | Y | N | 0 | -1.66678 | -4.03253 | 0.69898 | 0.218 | ns |
|  | 3 | | data.xe$VME_YMN | M | N | 0 | -1.4222 | -2.76775 | -0.07664 | 0.0359 | * |
|  |  | |  |  |  |  |  |  |  |  |  |
|  |  | | term | group1 | group2 | null.value | estimate | conf.low | conf.high | p.adj | p.adj.signif |
| Anthoathecata |  | | <chr> | <chr> | <chr> | <dbl> | <dbl> | <dbl> | <dbl> | <dbl> | <chr> |
|  | 1 | | data.st$VME_YMN | Y | M | 0 | -2.96021 | -5.27243 | -0.64799 | 0.00829 | ** |
|  | 2 | | data.st$VME_YMN | Y | N | 0 | -4.16854 | -6.62632 | -1.71076 | 0.000315 | *** |
|  | 3 | | data.st$VME_YMN | M | N | 0 | -1.20833 | -3.92125 | 1.504589 | 0.541 | ns |
